# Transcripts within rod photoreceptors of the Zebrafish retina

**DOI:** 10.1186/s12864-018-4499-y

**Published:** 2018-02-08

**Authors:** Chi Sun, Carlos Galicia, Deborah L. Stenkamp

**Affiliations:** Department of Biological Sciences, University of Idaho, 875 Perimeter Drive, MS 3051, Moscow, ID 83844-3051 USA

**Keywords:** Rod photoreceptor, Retina, Transcriptome, RNA-Seq, Zebrafish, Regeneration

## Abstract

**Background:**

The purpose of this study was to identify transcripts of retinal rod photoreceptors of the zebrafish. The zebrafish is an important animal model for vision science due to rapid and tractable development, persistent neurogenesis of rods throughout the lifespan, and capacity for functional retinal regeneration.

**Results:**

Zebrafish rods, and non-rod retinal cells of the *xops:eGFP* transgenic line, were separated by cell dissociation and fluorescence-activated cell sorting (FACS), followed by RNA-seq. At a false discovery rate of < 0.01, 597 transcripts were upregulated (“enriched”) in rods vs. other retinal cells, and 1032 were downregulated (“depleted”). Thirteen thousand three hundred twenty four total transcripts were detected in rods, including many not previously known to be expressed by rods. Forty five transcripts were validated by qPCR in FACS-sorted rods vs. other retinal cells. Transcripts enriched in rods from adult retinas were also enriched in rods from larval and juvenile retinas, and were also enriched in rods sorted from retinas subjected to a neurotoxic lesion and allowed to regenerate. Many transcripts enriched in rods were upregulated in retinas of wildtype retinas vs. those of a zebrafish model for rod degeneration.

**Conclusions:**

We report the generation and validation of an RNA-seq dataset describing the rod transcriptome of the zebrafish, which is now available as a resource for further studies of rod photoreceptor biology and comparative transcriptomics.

**Electronic supplementary material:**

The online version of this article (10.1186/s12864-018-4499-y) contains supplementary material, which is available to authorized users.

## Background

Within the vertebrate neural retina, photoreceptor cells are the sensory neurons that detect photons and convert this physical information into electrochemical signals. Rod photoreceptors contain the visual pigment rhodopsin, are highly sensitive to light, and provide predominantly convergent information to downstream neurons to maximize light detectability in low-light situations. Cone photoreceptors contain cone visual pigments (cone opsins) with distinct peak spectral sensitivities, and provide convergent and divergent information to downstream neurons, which process differential input to discriminate color and provide high acuity vision. Photoreceptors display distinctive morphologies with specialized apical projections, the outer segments, which are highly modified nonmotile cilia [1]. Outer segments include membranous disks to increase surface area for containing opsins and other phototransduction proteins, and photoreceptors maintain these outer segments with a high rate of protein synthesis, together with mechanisms for selective protein targeting and trafficking [2]. Rod photoreceptors in humans are particularly sensitive to genetic changes in structural and functional components; such defects cause hereditary retinal degenerations, which typically involve rod cell death, followed by cone cell death and loss of vision [3]. There is therefore great interest in increasing our depth of understanding of rod photoreceptor biology, health, the factors leading to cell death, and the discovery of strategies for promoting rod survival and/or rod replacement.

The zebrafish, an important animal model in vision research, is an example of a vertebrate with the endogenous capacity for rod replacement [4]. The zebrafish retina grows throughout its lifespan through the addition of new neurons at the retinal periphery, called the circumferential germinal zone (CGZ) or ciliary marginal zone (CMZ) [5–7]. In addition, Müller glia throughout the growing retina divide at a slow rate, generating a transiently-amplifying population of rod progenitors that migrate to the photoreceptor layer, and divide to generate rod photoreceptors [8]. The zebrafish retina therefore accumulates rods over its lifespan from these dedicated rod lineages. In zebrafish models of rod degeneration, either the most immediate precursors within the photoreceptor layer accelerate the production of new rods [9, 10], or the progenitor lineage is stimulated to increase rod neurogenesis to replace rods lost to damage [10]. In response to more widespread retinal damage due to chemical trauma, the progenitors generated by cell division of Müller glia gain the capacity to regenerate other types of retinal neurons [11–14], ultimately resulting in restoring visual function [11, 15]. The existence of the rod lineage is well-documented in zebrafish [8, 16, 17], and in other teleosts [18–20], and holds promise to inform the development of rod replacement strategies to treat human retinal disease. However, our knowledge of rods, and the rod lineage, within the zebrafish remains limited to a small number of rod-specific markers (primarily phototransduction components) [21], and a network of transcription factors important for rod determination and differentiation [5, 16, 22–25]. A single, distinctive marker for cells of the dedicated lineage that generates new rods, other than incorporation of S-phase markers, remains surprisingly elusive.

In the present study, we begin to fill this knowledge gap through RNA-sequencing (RNA-seq) analysis of the transcriptome of isolated rod photoreceptors, in comparison with non-rod retinal cells. In the transgenic line *xops:eGFP*, rod photoreceptors exclusively express high levels of GFP [26], permitting enrichment of rods from other retinal cells by fluorescence-activated cell sorting (FACS). This approach revealed transcripts that were upregulated in rods vs. other retinal cells, those that were present in rods but not differentially expressed, and those that were downregulated in rods vs. other retinal cells. Quantitative PCR (qPCR) studies suggested that this transcriptome is remarkably stable over the zebrafish lifespan from larval to adult ages, and appeared similar in rods that had regenerated following a chemical lesion. The zebrafish rod transcriptome is now a resource that can be mined for the identification of novel structural and functional components of rods, and possibly their progenitors, and for future comparative analyses with transcriptomes of rods and/or cones from key model organisms.

## Methods

### Animals and tissue preparation

All procedures involving animals were carried out in compliance with protocols approved by the University of Idaho Animal Care and Use Committee. Zebrafish (*Danio rerio*) were maintained on a 14:10 light:dark cycle in recirculating, monitored system water, housed and propagated according to [27]. For this study we used the *xops:eGFP* transgenic line, in which the *Xenopus* rod opsin promoter drives expression of eGFP exclusively in rod photoreceptors [26], the gift of James Fadool, and a wild-type strain originally obtained from Scientific Hatcheries (now Aquatica Tropicals). In addition we used the *xops:mCFP* transgenic line, the gift of Ann Morris. In this line, the presence of mCFP in retinal rods leads to rapid rod degeneration, and a proliferative response to this degeneration by the rod precursor population [9].

To obtain retinal tissues for fluorescence-activated cell sorting (FACS), *xops:eGFP* fish were dark-adapted for 10–12 h to facilitate removal of retina from the RPE, anaesthetized with MS-222, and eyes enucleated with fine forceps. Corneas and lenses were removed, and retinas were peeled free from the RPE and whole eyecup in saline. In some cases, as indicated in Results, we used whole adult (1.5 yrs), juvenile (1 month), or larval (14 days post-fertilization; dpf) retinas for FACS and quantitative RT-PCR (qPCR). In all cases, animals were dark-adapted prior to retina removal, and in all cases, RNA isolation was performed immediately following tissue collection or FACS.

Tissues for in situ hybridization were fixed in phosphate-buffered, 4% paraformaldehyde containing 5% sucrose for 1 h at room temperature, and washed in phosphate-buffered 5% sucrose, and then a graded series ending in 20% sucrose for overnight cyroprotection at 4 °C. Tissues were embedded in a 1:2 solution of OCT embedding medium (Sakura Finetek) and phosphate-buffered, 20% sucrose, and frozen in isobutane supercooled with liquid N_2_. After freezing solid, tissues were sectioned at 5 μm on a Leica CM3050 cryostat [15, 28].

### Cell dissociation and FACS

Whole retinas were dissociated into cell suspensions by incubating with 0.225% trypsin (Fisher ThermoScientific) and 0.001% papain (Worthington Biochemical) for 10 min at 37 °C. Dissociation was stopped by the addition of heat-inactivated fetal bovine serum (10% *v*/v final concentration). Suspended cells were pelleted and incubated with DNAseI at room temperature for 15 min. Cells were pelleted and resuspended in 100 μL phosphate-buffered (pH 6.5) saline (PBS) and immediately FACS-sorted.

GFP+ vs. GFP- retinal cells were sorted using a BD FACSAria flow cytometer, using the 488 nm laser and FITC fluorescence filter, and the 70 μm nozzle. Some cells were collected for fluorescence microscopy, or for post-sort FACS analysis. For RNA-seq or qPCR, GFP+ and GFP- cells were collected separately in the FACS sheath fluid, and RNA was immediately extracted.

### RNA isolation and quantitative real-time PCR (qPCR)

RNA was extracted from tissue samples using the NucleoSpin® RNA kit (Macherey-Nagel) using the manufacturer’s protocol, quantified and quality-checked on a Nanodrop spectrophotometer, and cDNA was synthesized using the SuperScript® kit (New England Biotech) using random hexamer primers. Gene-specific primers used for qPCR were designed using AlleleID7/84 (Premier Biosoft), and are provided in Table 1. Amplification was carried out using a model 7900HT Fast Real-Time PCR System and SYBR-Green PCRMaster Mix (Applied Biosystems, Inc.). Relative quantitation of gene expression between GFP+ and GFP- samples was determined using the 2^-ΔΔCt method with 18S as the reference transcript [29], and five technical replicates per biological replicate. For samples from adult fish, retinal cells from a single adult fish constituted a biological replicate (3–5 replicates); for samples from juvenile fish, retinal cells from 10 to 15 juveniles were pooled for each biological replicate (3 replicates); for samples from larvae, retinal cells from 15 to 20 larvae were pooled for each biological replicate (3 replicates).

**Table 1:** Primers used for qPCR

Gene	Sense Primer 5′ - > 3′	Anti-sense Primer 5′- > 3′
aipl1	TCCAGTCAGTCTTTACAC	CCTTAGTTCCAGTCACAA
ajap1	GGAGTAAGGTGTCTAACT	TTCCTGATATTCGTCCAT
apoc1l	CCCAATTACCTTGTGTTT	ACAGTGTGACTTTGTATTG
atat1	CTAATGTGAATCTGCTATA	ACTCAAGTTACTATCCAA
bbs4	ACCACATTAGGACTGCTG	TCATAGGTCAGAGCGTTTC
cabp4	AGTTCGTTATGATGTTGTCTCT	CTATGATGATCCGCCACTG
cobl	TCTAACCATACAGCAGAATCCA	GTCCAGGCGACAACATTG
cry3	TTACTCTTCTGGATTTCC	ATATAAACACACCGTACA
dscamb	AAGAAGATGGTCTGACTC	CAAGGGAAAGCAAGTATT
egf	TAAGTGAGTGGACAATGTT	GTCTTCGTGTTCCATCTA
enc1	ACGAGTCAGTATATTTCT	GTAAGTAACGAGCCTATA
esrrb	CGTCTCCTCATACTTCAG	TCCTCCACTCTATTAGCA
esrrd	CATGACCTTATGTGACCTT	CAGAAACCTGGTATGTGT
gc2	CTGTGTTAATTGGTGGAA	AGAGTATCGTAGGACATAA
gc3	CTCTATTCACTGCCATAT	CATGGTTACTGTTAAGAC
gngt1	AATCCATTCATTCAACACAACAT	ACTTCCATCTTCGCCTTATC
gucy2f	TAGCATTACACTATGGATT	GCCTATGATTCCTACTTT
kcnv2a	GCAGGAGTTAAGTAAGGATAT	TAGGAGTGGAGAACAGTC
kita	AATAAGCTTGCCGCCACCATGGAATATCACTGCGTTCT	CAAATATTTGTAGGTGAGCACAATCAGGATGAGAAC
lingo1a	TGCTTGTACGGATTGAAT	ATGTTGAGGAAACGAAGA
Lplastin	GCAGTGGGTGAACGAAACAC	TCGAGATCGCATACTTGGCG
mef2ca	TGTAATCATTCAGCGTAGTG	TCTAAGGTGTGCCGTTAT
mef2cb	CCCGTGAATAACCAGATC	GTGACATGCTGTTTCTTT
mpeg1.1	CGGGTTCAAGTCCGTAACCA	TGGCGTCAGCGATTTCTTCT
msi1	CGAGCCCAGCCTAAGTTG	ATCTTCAATAGTCGTGTTCACTGA
ncam1b	AGTTTGATAAAGATGTTCGTTTC	TTAATGCTGCGGAAGTCA
ngf	GAGAAGACTACAAGCGAAT	CGACAACAATAAGGAGGAT
nr1d4a	AATCATCTTATCGCACAAC	ATAGTAGTAGGTAGTAGGAGTA
nr1d4b	AACGGTCACTATAACTTC	GAATAGCTGTTGTGTTTAG
nr2f1b	TGAGAAGAACACAGAGTAA	AGGATTGCTGACTATAACA
nrl	GATGGTCAGAGGAGAATG	GGTTGTAACGAGTGCTTA
nucb2b	ATGATATGGTGGAGATGGA	CTTGTTCGTGGCAGTAAT
panx1b	GCAGAGTGATTCTAAGTA	GAGTGAGATGAGTAACAA
pdca	TGCCGATGTGGAATAATCAGA	ACAGCGTCATTACTCATTCTATCT
ppdpfa	TAGCGTTTACCCGACCAA	TTTCCCCGTCCTCTAAAG
prom1b	CAGTTGGAGTGACAGTTG	TCAGGTCTCTTATGTTGGT
rtn2a	GGACACATAGACACAGACAA	CCTTCCAGTAGACCAGGT
rho	ACTTCCGTTTCGGGGAGAAC	GAAGGACTCGTTGTTGACAC
rhol	GCTGTGAGATGCTGGATT	GTTGTTGTTGTTGTTGTTGTC
rims3	AGAGGAGGTCAGTTAGAG	TATATGTTGCTGGAATGTTC
rxrga	TTCACACTGGTCATTCAA	AAGGCATTATAGAGCGATT
rxrgb	ACATAATACAGACAGAGACT	TAATAGCACAAGACAGAATC
thrb	TCTGGTCTGATGAGTCTA	GTATTAGCCTGGTGATGA
tprn	CAAACAACAAACATATAATCAAGT	TCTGAATGGTCGTGAATG
tulp1b	CAAGGAATCAACAGAGAAG	CATCATCATCATCGTCATC
sept8b	CTATCGTGGACTACATTGA	ATGAAGTACAGGCAGATG
znf536	CAATGGACAGAATTTAGGAATCA	CACAAAGAGGACAGGGATAT
18S	GAACGCCACTTGTCCCTCTA	GTTGGTGGAGCGATTTGTCT

### Library construction, RNA-seq, and bioinformatics

**Fig. 1:**
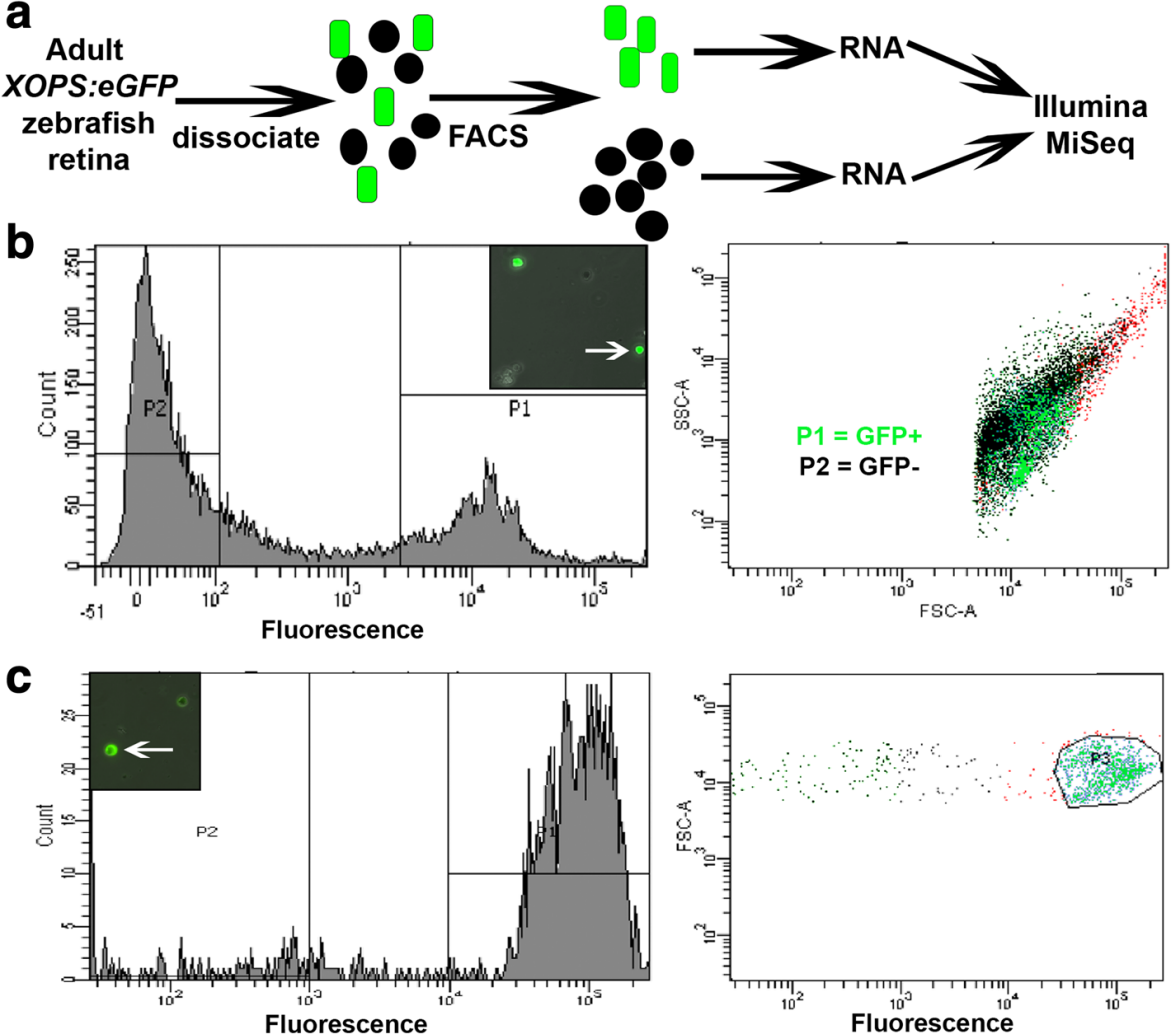
Dissociation, FACS separation, and RNA-seq analysis of GFP+ rod photoreceptors and GFP- retinal cells. **a** Experimental procedure. **b** Representative FACS results showing dissociated cells (inset; arrow indicates GFP+ cell), GFP+ population collected in P1, GFP- population collected in P2. Cells not in P1 or P2 are in red in the second panel. **c** Post-sort analysis of a sorted GFP+ population by fluorescence microscopy (inset; arrow indicates GFP+ cell) and FACS. SSC, side scatter (reflecting object complexity); FSC, forward scatter (reflecting object size)

Both quantity and quality of RNA were assessed by using an Agilent 2100 Bioanalyzer. All samples used for RNA-seq had an RNA integrity number (RIN) > 8.0, and the experimental design retained pairing information between GFP+ cells and GFP- cells derived from both retinas of a single fish, allowing us to analyze them as paired samples. At least 5 ng of RNA was available per sample, and provided to the University of Idaho’s Institute for Bioinformatics and Evolutionary Studies (IBEST) Genomics Core for RNA amplification, the generation of cDNA, sequencing, and bioinformatics. Quality and quantity of cDNA libraries were verified by Bioanalyzer. All sample preparation was achieved with Ovation® RNA-Seq System V2 (NuGEN), and sequencing performed on an Illumina (San Diego, CA) MiSeq with MiSeq Reagent Kit v3, 600 cycle kit. Four biological replicates (from four different fish) were sequenced (Fig. 1a). Reads were quality-trimmed with Sickle (https://github.com/najoshi/sickle), and paired reads were overlapped with FLASH [30]. Overlapped reads were aligned against Zv9.75 using the Burrows-Wheeler aligner [31]. BAM files were sorted with using SAMtools [32], and reads were counted by feature using HTSeq-count [33]. Counts were analyzed and differentially expressed genes were identified with R [34] and edgeR [35]. Descriptive plots were generated, and gene ontology (GO) analysis and hierarchical clustering were performed, with R and GOstats [36]. Comparison with a publicly available microarray dataset [37] was done using paralogue and probe identifier information available via Ensembl’s BioMart (http://uswest.ensembl.org/biomart/martview/83d09c7c5ff71f36d9df58ed9f566c78).

### Probe preparation and in situ hybridization

Zebrafish *rho* (*rhodopsin*) cDNA, in pBK-CMV phagemid, was the gift of T. Vihtelic. Other cDNAs were generated as follows. Total RNA was extracted from homogenized adult zebrafish retina, and cDNA was generated using random hexamers and oligo(dT) primers. Gene-specific primers (Table 2) corresponding to *rhol*, *dscamb*, *rxrga*, and *rxrgb* predicted mRNAs were designed using Primer-BLAST (https://www.ncbi.nlm.nih.gov/tools/primer-blast/), were used for PCR amplifications, and the resulting amplicons were gel-purified and ligated using TA-ligation into the pGEM-T-Easy vector (Promega), which contains T7 and SP6 promoters. cDNAs were sequence-verified (*ElimBio;* St. Hayward, CA), with sequencing results compared to original genomic sequence using nucleotide Blast software and viewed in *Sequencher* (GeneCodes). Digoxigenin (dig) –labeled cRNA probes were prepared using T7 or SP6 RNA polymerase (Roche) according to the manufacturer’s instructions. In situ hybridization was carried out according to Nelson et al. [16]. In brief, sections were rehydrated, permeabilized with proteinase K, dehydrated and incubated with probe in a solution containing 50% formamide, with hybridization temperatures optimized for each probe using PolyPro [38]. Hybridized tissues were treated with RNAse A, and the presence of dig was detected with anti-dig antibodies conjugated to alkaline phosphatase, followed by an NBT-BCIP (Roche) or BM-purple (Sigma) color reaction carried out according to the manufacturer’s instructions. *In situs* were imaged on a Leica DM2500 upright microscope with a Leica DFC700T camera using DIC optics. In addition to antisense probes, sense probes were also prepared and confirmed not to generate detectable signal.

**Table 2:** Primers used for generation of in situ probes

Primer name	Primer sequence 5′- > 3′	Probe length
rhol_probe_F	CGAAGTGACCCGAATGGTGA	764b
rhol_probe_R	GCGGAATGAACCGCCTTAAC	
dscamb_probe_F	TCTGGATCCCCGGAGACAAT	757b
dscamb_probe_R	TCTGGATCCCCGGAGACAAT	
rxrga_probe_F	GGAGAAGATCCTGGACGCTG	734b
rxrga_probe_R	AGTGTGCGCTGGGGTTTATT	
rxrgb_probe_F	CGCGGAATGGATACTCACGA	807b
rxrgb_probe_R	TCCGCTGCATGGCAGATATT	

### Retinal damage and regeneration

The retinas of adult *xops:eGFP* fish (1 yr) were chemically lesioned to destroy all retinal neurons while sparing Müller glia [13]. Briefly, fish were anaesthetized by tricaine and an incision was made across the cornea with a sapphire blade. Using a Hamilton syringe, 0.4 μL - 0.6 μL of 200 μM ouabain was injected into the vitreal chamber of the right eye, resulting in an estimated intraocular concentration of 10 μM [11, 15, 39]. Loss of GFP+ photoreceptors was verified in sectioned retinas obtained from parallel experiments at 3 days post-injury (3 dpi), and by viewing retinas of live, anaesthetized fish with epifluorescence stereomicroscopy (Leica M165 FC), also at 3 dpi. Lesioned zebrafish were allowed to recover, and regenerate their retinas [11] until 14 dpi or 30 dpi, and were humanely sacrificed to collect retinas for cell dissociation, FACS, and qPCR, or to collect whole eyes for cryosectioning.

### Indirect immunofluorescence and confocal microscopy

Histological processing and sectioning was carried out as previously described [28]. In brief, whole eyes were perforated at the cornea, to create a slit through which lenses were removed. Eyecups were immersed in phosphate-buffered (pH -7.4), 4% paraformaldehyde containing 5% sucrose and fixed at room temperature for 1 h. Eyes were subjected to sequential washes with increasing concentrations of phosphate-buffered sucrose, and cryoprotected overnight at 4 °C in phosphate-buffered, 20% sucrose. Eyes were embedded in a 1:2 mixture of OCT (optimal cutting temperature; Sakura Finetek) and buffered, 20% sucrose, and sectioned on a Leica CM3050 cryostat at 5 μm. Sections were blocked in 20% goat serum 1 h, room temperature, and then incubated with primary antibody 1D1 (1:20) (the gift of James Fadool) overnight, 4 °C, which stains zebrafish rhodopsin [40]. Sections were washed with phosphate-buffered (pH -7.4) saline containing 0.01% TritonX-100 (PBST), and then incubated with secondary antibody conjugated to Cy3 (1:200; Jackson Immunoresearch) for 2 h at room temperature. Mounted sections were imaged with a Nikon Andor spinning disk confocal microscope and Zyla sCMOS camera using 40X dry and 60X oil immersion objectives.

## Results

### Transcripts of rods of adult zebrafish retina

We isolated highly pure rod and non-rod retinal cell populations by FACS-sorting cell suspensions from adult *xops:eGFP* zebrafish retinas (Fig. 1a). GFP+ cells (P1 in Fig. 1b) made up 10–20% of all collected retinal cells and constituted a distinctive cell population as compared with the GFP- population (P2 in Fig. 1b). Visualization of samples using epifluorescence microscopy suggested that the majority of dissociated GFP+ profiles, and FACS-sorted GFP profiles, appeared to be rounded-up rod cell bodies rather than outer segments or other fragments (insets in Fig. 1b, c). To verify enrichment of our sorted populations, a separate sample from one fish was used to collect GFP+ cells using the same FACS-sorting parameters as those used for RNA-seq, and we subsequently examined the sorted population by fluorescence microscopy and by post-sort analysis (Fig. 1c). These results indicated that P1 population was highly enriched for GFP+ cells (Fig. 1c), and therefore suitable for transcriptome analysis.

We performed RNA-seq on both the rod (GFP+; P1) and the “non-rod” (GFP-; P2) populations, in order to identify transcripts enriched, present, or depleted in the rod population as compared with other retinal cells (similar to the approach of [41]). The resulting dataset is publicly available via the Gene Expression Omnibus (GEO; accession # GSE100062). Sequencing depth ranged from 2,781,516 to 3,480,515 reads per sample, and mapping percentages ranged from 97.0% to 97.4% per sample. Multidimensional scaling showed good separation of GFP+ vs. GFP- samples along the first dimension and separation by sample along the second dimension (Additional file 1: Figure S1A). A plot of estimated Biological Coefficient of Variation (BCV) indicated a trend in dispersion associated with expression (Additional file 1: Figure S1B), leading us to fit a trended model within edgeR before doing differential expression analysis. Differentially expressed transcripts were identified as those significantly upregulated or downregulated in the GFP+ population vs. the GFP- population. Those identified with a false discovery rate (FDR) of < 0.01 consisted of 1629 distinct entries (597 upregulated, 1032 downregulated); those identified with an FDR of < 0.05 consisted of 2439 entries (Additional file 1: Figure S1C). The top 50 upregulated and top 50 downregulated transcripts within the GFP+ vs. GFP- populations, based upon FDR, are provided as Tables 3 and 4, respectively. Numerous transcripts known to be expressed exclusively by rods were significantly upregulated (enriched) in the GFP+ cell population, including *rho*, *pde6g*, *rom1b*, and *gnat1* (Table 3). Numerous transcripts known to be primarily expressed by other retinal cell types were significantly downregulated (depleted) in the GFP+ cell population, including cone transcripts *opn1lw2* (long wavelength-sensitive cone opsin 2), *opn1mw3* (cone opsin *rh2–3*), and *cnga3a*, and the macrophage/microglial marker *mpeg1* [42] (Table 4). These outcomes further corroborated the rod (GFP+) vs. non-rod (GFP-) identities of our sample cell populations.

**Table 3:** Top 50 transcripts significantly upregulated (enriched) in GFP+ (rods) vs GFP- retinal cell populations

Name	Description	logFC	FDR
gc2	guanylyl cyclase 2 [Source:ZFIN;Acc:ZDB-GENE-011128-8]	3.032892	5.22E-22
esrrd	estrogen-related receptor delta [Source:ZFIN;Acc:ZDB-GENE-040616-3]	3.642435	8.52E-17
zgc:112,334	zgc:112,334 [Source:ZFIN;Acc:ZDB-GENE-050809-120]	3.829432	3.64E-16
gngt1	guanine nucleotide binding protein (G protein), gamma transducing activity polypeptide 1 [Source:ZFIN;Acc:ZDB-GENE-030131-7596]	2.959265	4.60E-16
arhgap29a	Rho GTPase activating protein 29a [Source:ZFIN;Acc:ZDB-GENE-030131-9510]	2.653423	8.20E-16
kitb	kit receptor b [Source:ZFIN;Acc:ZDB-GENE-050916-2]	2.782837	1.06E-15
si:dkey-204f11.59	si:dkey-204f11.59 [Source:ZFIN;Acc:ZDB-GENE-040724-220]	3.212857	2.27E-15
ajap1	adherens junctions associated protein 1 [Source:ZFIN;Acc:ZDB-GENE-041210-353]	2.499397	7.05E-15
OSBPL1A (2 of 2)	oxysterol binding protein-like 1A [Source:HGNC Symbol;Acc:16,398]	2.427894	7.09E-15
pde6g	phosphodiesterase 6G, cGMP-specific, rod, gamma [Source:ZFIN;Acc:ZDB-GENE-030904-1]	3.045885	9.01E-15
UBAP1L (1 of 2)	ubiquitin associated protein 1-like [Source:HGNC Symbol;Acc:40,028]	2.868683	9.01E-15
rcvrna	recoverin a [Source:ZFIN;Acc:ZDB-GENE-050913-106]	2.719941	5.43E-14
rom1b	retinal outer segment membrane protein 1b [Source:ZFIN;Acc:ZDB-GENE-040426-1073]	2.85896	5.78E-14
rorb	RAR-related orphan receptor B [Source:ZFIN;Acc:ZDB-GENE-061204-2]	2.48207	6.19E-14
tmtops2a	teleost multiple tissue opsin 2a [Source:ZFIN;Acc:ZDB-GENE-130129-3]	2.265541	1.05E-13
cerkl	ceramide kinase-like [Source:ZFIN;Acc:ZDB-GENE-070410-38]	2.181324	1.11E-13
cobl	cordon-bleu homolog (mouse) [Source:ZFIN;Acc:ZDB-GENE-091020-11]	2.417617	1.19E-13
hcn3	hyperpolarization activated cyclic nucleotide-gated potassium channel 3 [Source:ZFIN;Acc:ZDB-GENE-060503-193]	3.197947	1.51E-13
zgc:162,144	zgc:162,144 [Source:ZFIN;Acc:ZDB-GENE-030131-7630]	2.775518	1.70E-13
gnb1b	guanine nucleotide binding protein (G protein), beta polypeptide 1b [Source:ZFIN;Acc:ZDB-GENE-040426-2855]	2.767042	1.70E-13
unc119.2	unc-119 homolog 2 [Source:ZFIN;Acc:ZDB-GENE-030131-7635]	2.116224	1.79E-13
PTPDC1 (1 of 3)	protein tyrosine phosphatase domain containing 1 [Source:HGNC Symbol;Acc:30,184]	3.253732	2.91E-13
pdca	phosducin a [Source:ZFIN;Acc:ZDB-GENE-031023-1]	2.797727	3.06E-13
rom1a	retinal outer segment membrane protein 1a [Source:ZFIN;Acc:ZDB-GENE-040426-1765]	3.176656	3.68E-13
kcnv2a	potassium channel, subfamily V, member 2a [Source:ZFIN;Acc:ZDB-GENE-091117-27]	2.625431	4.38E-13
rho	rhodopsin [Source:ZFIN;Acc:ZDB-GENE-990415-271]	3.214731	4.63E-13
cplx4c	complexin 4c [Source:ZFIN;Acc:ZDB-GENE-101018-1]	2.863052	7.98E-13
samd11	sterile alpha motif domain containing 11 [Source:ZFIN;Acc:ZDB-GENE-060428-2]	2.708617	9.40E-13
BX248120.1	Uncharacterized protein [Source:UniProtKB/TrEMBL;Acc:E7F7S5]	2.586515	1.34E-12
saga	S-antigen; retina and pineal gland (arrestin) a [Source:ZFIN;Acc:ZDB-GENE-040426-1538]	3.089512	1.44E-12
arhgef10lb	Rho guanine nucleotide exchange factor (GEF) 10-like b [Source:ZFIN;Acc:ZDB-GENE-090313-222]	2.476543	1.60E-12
guca1a	guanylate cyclase activator 1A [Source:ZFIN;Acc:ZDB-GENE-011128-5]	2.765826	1.68E-12
si:dkeyp-41f9.3	si:dkeyp-41f9.3 [Source:ZFIN;Acc:ZDB-GENE-091118-56]	2.849587	1.72E-12
TDRD7B	Tudor domain-containing protein 7B [Source:UniProtKB/Swiss-Prot;Acc:E7FDW8]	2.840789	2.06E-12
PLCH2 (1 of 2)	phospholipase C, eta 2 [Source:HGNC Symbol;Acc:29,037]	2.020111	2.30E-12
cabp4	calcium binding protein 4 [Source:ZFIN;Acc:ZDB-GENE-081104-291]	2.513244	2.77E-12
guca1b	guanylate cyclase activator 1B [Source:ZFIN;Acc:ZDB-GENE-011128-6]	2.703103	2.78E-12
grk1a	G protein-coupled receptor kinase 1 a [Source:ZFIN;Acc:ZDB-GENE-050823-1]	2.930581	1.10E-11
gnb1a	guanine nucleotide binding protein (G protein), beta polypeptide 1a [Source:ZFIN;Acc:ZDB-GENE-030131-823]	2.886447	1.19E-11
slc6a15	solute carrier family 6 (neutral amino acid transporter), member 15 [Source:ZFIN;Acc:ZDB-GENE-050420-93]	2.779643	1.45E-11
ppdpfa	pancreatic progenitor cell differentiation and proliferation factor a [Source:ZFIN;Acc:ZDB-GENE-030219-204]	2.669857	1.71E-11
pde6b	phosphodiesterase 6B, cGMP-specific, rod, beta [Source:ZFIN;Acc:ZDB-GENE-090421–2]	3.049742	2.01E-11
znf536	zinc finger protein 536 [Source:ZFIN;Acc:ZDB-GENE-030616-624]	2.683055	2.19E-11
SUSD3	sushi domain containing 3 [Source:HGNC Symbol;Acc:28,391]	2.124055	2.28E-11
gnat1	guanine nucleotide binding protein (G protein), alpha transducing activity polypeptide 1 Source:ZFIN;Acc:ZDB-GENE-011128-11]	2.967409	3.45E-11
sagb	S-antigen; retina and pineal gland (arrestin) b [Source:ZFIN;Acc:ZDB-GENE-050913-98]	3.155676	3.69E-11
asmt	acetylserotonin O-methyltransferase [Source:ZFIN;Acc:ZDB-GENE-080220-43]	1.844975	4.41E-11
pfkfb4l	6-phosphofructo-2-kinase/fructose-2,6-biphosphatase 4, like [Source:ZFIN;Acc:ZDB-GENE-031031-4]	1.93027	4.63E-11
alpl	alkaline phosphatase, liver/bone/kidney [Source:ZFIN;Acc:ZDB-GENE-040420-1]	2.701265	5.22E-11
slc24a1	solute carrier family 24 (sodium/potassium/calcium exchanger), member 1 [Source:ZFIN;Acc:ZDB-GENE-060503-191]	2.88482	7.45E-11

**Table 4:** Top 50 transcripts significantly downregulated (depleted) in GFP+ (rods) vs GFP- retinal cell populations

Name	Description	logFC	FDR
si:dkey-27i16.2	si:dkey-27i16.2 [Source:ZFIN;Acc:ZDB-GENE-030131-9667]	−6.03866537	1.88E-38
ptprc	protein tyrosine phosphatase, receptor type, C [Source:ZFIN;Acc:ZDB-GENE-050208-585]	−6.06476722	3.46E-31
apoc1l	apolipoprotein C-I like [Source:ZFIN;Acc:ZDB-GENE-030131-1074]	−4.46320866	2.75E-30
cd74a	CD74 molecule, major histocompatibility complex, class II invariant chain a [Source:ZFIN;Acc:ZDB-GENE-000901-1]	−4.67633439	1.65E-28
bzw1b	basic leucine zipper and W2 domains 1b [Source:ZFIN;Acc:ZDB-GENE-040426-2881]	−3.66358803	9.06E-28
si:dkey-25o1.6	si:dkey-25o1.6 [Source:ZFIN;Acc:ZDB-GENE-091204-276]	−5.26240739	3.27E-27
si:ch211–260d11.1	si:ch211–260d11.1 [Source:ZFIN;Acc:ZDB-GENE-091204-40]	−6.02459793	1.05E-26
lgals3bpb	lectin, galactoside-binding, soluble, 3 binding protein b [Source:ZFIN;Acc:ZDB-GENE-040426-2262]	−5.09075533	1.25E-26
coro1a	coronin, actin binding protein, 1A [Source:ZFIN;Acc:ZDB-GENE-030131-9512]	−7.62303025	1.06E-25
hbaa1	hemoglobin alpha adult-1 [Source:ZFIN;Acc:ZDB-GENE-980526-79]	−9.74873113	1.70E-25
si:dkey-25o1.5	si:dkey-25o1.5 [Source:ZFIN;Acc:ZDB-GENE-091204-344]	−9.53659867	7.14E-23
sla2	Src-like-adaptor 2 [Source:ZFIN;Acc:ZDB-GENE-080204-98]	−9.56172956	2.35E-22
inpp5d	inositol polyphosphate-5-phosphatase D [Source:ZFIN;Acc:ZDB-GENE-100922–30]	−4.44994274	1.17E-21
mpeg1	macrophage expressed 1 [Source:ZFIN;Acc:ZDB-GENE-030131-7347]	−9.35334321	2.15E-21
ZFP36	ZFP36 ring finger protein [Source:HGNC Symbol;Acc:12,862]	−5.4525006	1.53E-20
ankrd33ab	ankyrin repeat domain 33Ab [Source:ZFIN;Acc:ZDB-GENE-100729-1]	−3.13603996	1.97E-20
pfn1	profilin 1 [Source:ZFIN;Acc:ZDB-GENE-031002–33]	−4.86406116	2.72E-20
grk7a	G-protein-coupled receptor kinase 7a [Source:ZFIN;Acc:ZDB-GENE-050824-1]	−2.77617205	5.07E-20
rcv1	recoverin [Source:ZFIN;Acc:ZDB-GENE-030131-7590]	−2.80718642	2.57E-19
tagapa	T-cell activation RhoGTPase activating protein a [Source:ZFIN;Acc:ZDB-GENE-040426-1877]	−6.39333701	1.05E-18
si:dkey-126 g1.9	si:dkey-126 g1.9 [Source:ZFIN;Acc:ZDB-GENE-030131-9862]	−2.79689496	1.53E-18
havcr1	hepatitis A virus cellular receptor 1 [Source:ZFIN;Acc:ZDB-GENE-040718-131]	−7.67482315	1.91E-18
slc1a8b	solute carrier family 1 (glutamate transporter), member 8b [Source:ZFIN;Acc:ZDB-GENE-070912-552]	−2.5380374	3.55E-18
csf1ra	colony stimulating factor 1 receptor, a [Source:ZFIN;Acc:ZDB-GENE-001205-1]	−4.24059459	1.94E-17
si:ch211–250 g4.3	si:ch211–250 g4.3 [Source:ZFIN;Acc:ZDB-GENE-060503-506]	−7.55964405	3.53E-17
arpc1b	actin related protein 2/3 complex, subunit 1B [Source:ZFIN;Acc:ZDB-GENE-030131-7414]	−4.66326368	6.41E-17
CT826376.1	Uncharacterized protein [Source:UniProtKB/TrEMBL;Acc:E7F690]	−2.34407255	7.14E-17
pdcb	phosducin b [Source:ZFIN;Acc:ZDB-GENE-031023-2]	−2.4532108	9.01E-17
opn1lw2	opsin 1 (cone pigments), long-wave-sensitive, 2 [Source:ZFIN;Acc:ZDB-GENE-040718-141]	−2.76413479	5.15E-16
ba1	ba1 globin [Source:ZFIN;Acc:ZDB-GENE-990415-18]	−6.23069882	6.15E-16
opn1mw3	opsin 1 (cone pigments), medium-wave-sensitive, 3 [Source:ZFIN;Acc:ZDB-GENE-030728-6]	−2.56365947	9.40E-16
hbaa1	hemoglobin alpha adult-1 [Source:ZFIN;Acc:ZDB-GENE-980526-79]	−6.44638081	2.14E-15
zgc:195,245	zgc:195,245 [Source:ZFIN;Acc:ZDB-GENE-081022-200]	−2.62839443	2.47E-15
arr3a	arrestin 3a, retinal (X-arrestin) [Source:ZFIN;Acc:ZDB-GENE-040718-102]	−3.02133496	2.47E-15
zgc:100,919	zgc:100,919 [Source:ZFIN;Acc:ZDB-GENE-040718-248]	−4.70079878	3.07E-15
gc3	guanylyl cyclase 3 [Source:ZFIN;Acc:ZDB-GENE-011128-9]	−2.32749631	4.00E-15
ppp1r18	protein phosphatase 1, regulatory subunit 18 [Source:ZFIN;Acc:ZDB-GENE-060503-350]	−2.29809174	5.42E-15
CD68	CD68 molecule [Source:HGNC Symbol;Acc:1693]	−3.83135448	5.42E-15
il1b	interleukin 1, beta [Source:ZFIN;Acc:ZDB-GENE-040702-2]	−4.3585252	5.67E-15
cplx4a	complexin 4a [Source:ZFIN;Acc:ZDB-GENE-060526-116]	−2.12559816	7.51E-15
cnga3a	cyclic nucleotide gated channel alpha 3a [Source:ZFIN;Acc:ZDB-GENE-090611–2]	−2.2388126	1.69E-14
ccr9a	chemokine (C-C motif) receptor 9a [Source:ZFIN;Acc:ZDB-GENE-060130-125]	−4.13641441	1.75E-14
opn1lw1	opsin 1 (cone pigments), long-wave-sensitive, 1 [Source:ZFIN;Acc:ZDB-GENE-990604-41]	−2.8443546	1.89E-14
KEL	Kell blood group, metallo-endopeptidase [Source:HGNC Symbol;Acc:6308]	−8.54223593	2.27E-14
si:ch1073-403i13.1	si:ch1073-403i13.1 [Source:ZFIN;Acc:ZDB-GENE-100921–25]	−4.50874649	2.31E-14
wasb	Wiskott-Aldrich syndrome (eczema-thrombocytopenia) b [Source:ZFIN;Acc:ZDB-GENE-030131-7098]	−5.26157912	2.52E-14
SLC24A2 (1 of 2)	solute carrier family 24 (sodium/potassium/calcium exchanger), member 2 [Source:HGNC Symbol;Acc:10,976]	−2.22076034	2.67E-14
pbxip1b	pre-B-cell leukemia homeobox interacting protein 1b [Source:ZFIN;Acc:ZDB-GENE-070112-2032]	−2.27917445	3.27E-14
CD53	CD53 molecule [Source:HGNC Symbol;Acc:1686]	−5.72855472	3.28E-14
slc25a25a	solute carrier family 25 (mitochondrial carrier; phosphate carrier), member 25a [Source:ZFIN;Acc:ZDB-GENE-040426-2396]	−2.50025348	3.72E-14

**Fig. 2:**
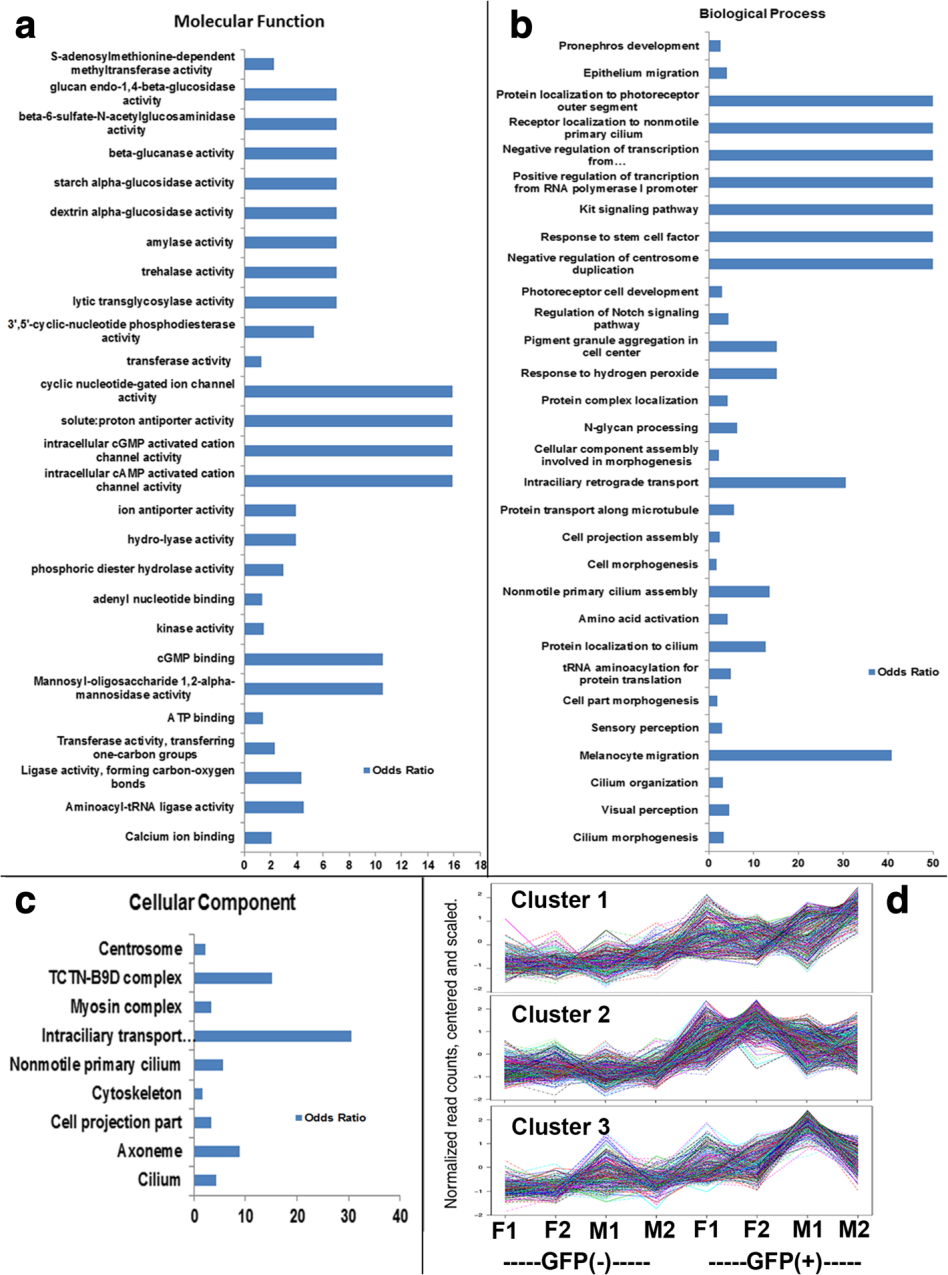
Gene ontology (GO) and hierarchical cluster analysis. **a-c** Molecular functions (**a**), biological processes (**b**), and cellular components (**c**) overrepresented in the GFP+ (rod photoreceptor) cell population (*p* < 0.01). **d** Hierarchical clustering of rod-enriched transcripts reveals only three, highly similar clusters. F, female; M, male

To provide broad classification of rod-enriched transcripts, we used gene ontology (GO) analysis. GO molecular function categories significantly overrepresented in GFP+ samples included those related to cyclic nucleotide metabolism, intermediary metabolism, and ion transport (Fig. 2a), and GO biological processes that were overrepresented included intracellular transport processes, photoreceptor cell development, and the kit and notch signaling pathways (Fig. 2b). GO biological process categories related to cell stress or apoptosis were not overrepresented, suggesting that, although the GFP+ rods did not maintain their morphologies during the dissociation and sorting procedures (insets in Fig. 1a, b), these procedures likely did not cause differential upregulation of genes related to stress and cell death in the rods. These findings are consistent with the recent report by Richardson et al. [43] showing that FACS does not perturb gene expression. GO cellular component categories significantly overrepresented in the GFP+ samples included those related to cytoskeletal components and those related to cilia (Fig. 2c). These categories reflect the underlying structure, function, and very likely the ongoing developmental programs engaged for maintaining rod structure and function. It was surprising, however, that some of these categories were overrepresented considering the large number of cones likely to be present in the GFP- retinal cell population that were also expected to demonstrate similar molecular functions, biological processes, and cellular components. Hierarchical clustering of the rod-enriched transcripts returned only three, highly similar clusters (Fig. 2d), suggesting very little sample heterogeneity within the GFP+ samples and within the GFP- samples. Our experimental design included sex as a potential biological variable, such that we could analyze a sex X rod interaction. This analysis returned only five entries with an FDR < 0.05 (Table 5).

**Table 5:** Sex X rod interaction

Name	Description	logFC	FDR	Comment
pmela	premelanosome protein a [Source:ZFIN;Acc:ZDB-GENE-030131-9818]	15.0198	6.05E-06	Absent in female rods; Present in male rods
slc6a6b	solute carrier family 6 (neurotransmitter transporter, taurine), member 6b [Source:ZFIN;Acc:ZDB-GENE-030131-3077]	−4.06708	3.31E-05	Enriched in female rods; depleted in male rods
naa35	N(alpha)-acetyltransferase 35, NatC auxiliary subunit [Source:ZFIN;Acc:ZDB-GENE-030131-306]	−5.25565	0.025774	Enriched in female rods; depleted in male rods
si:ch211-89f7.1	si:ch211-89f7.1 [Source:ZFIN;Acc:ZDB-GENE-060526-180]	−2.75147	0.025774	Enriched in female rods; less enriched in male rods
ush2a	Usher syndrome 2A (autosomal recessive, mild) [Source:ZFIN;Acc:ZDB-GENE-060503-794]	−2.65862	0.025941	Enriched in female rods; not enriched in male rods

The analyses described above focused upon transcripts that were differentially expressed in GFP+ vs. GFP- cells, therefore identifying enriched transcripts in either population. To identify additional transcripts present in rods, but not necessarily enriched in comparison with other retinal cells, we generated a list of transcripts for which all GFP+ samples returned a non-zero value. This list of transcripts present in rods amounted to 13,324 distinct entries (not shown), approximately 23% of the total number of predicted transcripts (58,549; Ensembl GRCz10) encoded by the zebrafish genome. This list included numerous photoreceptor (but not rod-specific) genes such as *irbp*, *neurod*, *crx*, and *rx1*, and some genes not previously known to be expressed in rod photoreceptors, such as *opsin 4.1*, a zebrafish *melanopsin* [44], and several nuclear hormone receptor genes including *rxrga*. The latter is noteworthy because the mouse orthologue (*RXRγ*) was reported to be cone-specific [45].

Forty-five transcripts were selected for validation analysis by qPCR in independently sorted GFP+ vs. GFP- cells from adult *xops:eGFP* zebrafish. Transcripts were prioritized for validation based upon predicted or know functions as transcription factors (e.g. *esrrb, nrl*, *nr2f1b*, *rxrga*), as components of cell signaling pathways (e.g. *ngf*, *kita*), or as cell adhesion molecules (e.g. *dscamb*, *ncam1b*), because future analysis of such components stands to reveal new insights into regulation of rod development, maturation, and/or maintainance. We also selected some transcripts known to have structural (e.g. *prom1b*, *bbs4*) or functional requirements in photoreceptors (e.g. *rhol*, *gngt1*, *pdca*). Finally, we selected transcripts that were detected as enriched in rods, as well as some that were detected as present in rods, but not enriched, to more thoroughly validate the RNA-seq dataset.

**Fig. 3:**
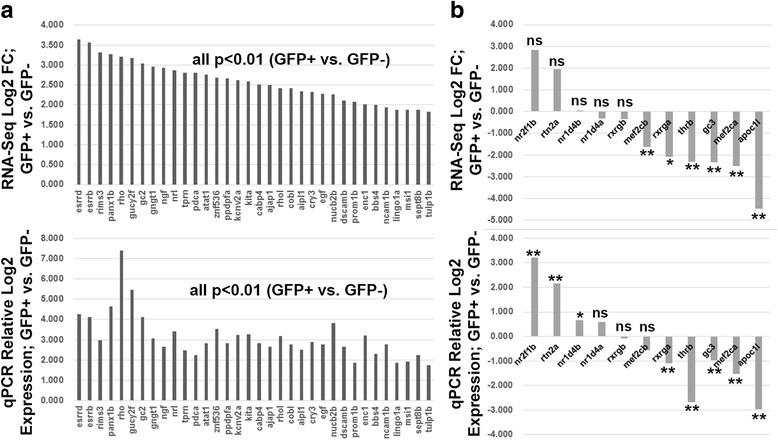
Quantitative PCR (qPCR) validation of transcripts enriched in GFP+ (rod photoreceptor) cells (**a**), and present or depleted in GFP+ cells (**b**). Top panel in each shows RNA-Seq results; bottom panel shows qPCR. **, p < 0.01; *, *p* < 0.05; ns, not significantly differentially expressed, for GFP+ vs. GFP- (three biological replicates)

Selected transcripts that were detected by RNA-seq as significantly enriched in rods, were all determined to be significantly enriched in rods by qPCR, at similar relative magnitudes (Fig. 3a). Selected transcripts that were detected by RNA-seq as present, though not enriched in rods, were all detectable by qPCR (see first five genes in Fig. 3b). Some of these were detected by qPCR to be significantly enriched (the first three genes in Fig. 3b), a minor discrepancy perhaps related to the larger number of biological replicates used for qPCR. Transcripts detected by RNA-seq as significantly depleted but still present in rods, were nearly all determined to be significantly depleted in rods by qPCR, with the exception of *mef2cb*, where qPCR did not detect a significant difference (see last six genes in Fig. 3b), but nevertheless detected the presence of this transcript in rods. These qPCR results provide strong validation that the RNA-seq dataset generated in this study will serve as a reliable resource for many future applications.

### Rod photoreceptor transcripts over the lifespan and in a genetic model for rod degeneration

As zebrafish grow, they continue to generate new rods throughout the retina from a dedicated rod lineage [4, 16], the apex of which (stem cell for rod lineage) has been identified as the Müller glial cell [8]. Therefore we wished to determine whether the rod transcriptome remained consistent over the zebrafish lifespan, or if the rod population of larval zebrafish would be distinct from the accumulated (and generally older) rod population of adult zebrafish. A subset of the selected transcripts that were qPCR validated in adult zebrafish, were therefore evaluated further by qPCR in GFP+ vs. GFP- retinal cell populations obtained from larval zebrafish sacrificed at 14 dpf (days post-fertilization), and juvenile zebrafish sacrificed at 30 dpf. Rod photoreceptors are considered morphologically mature at 15 dpf [46], but do not contribute to adult-like scotopic (rod-driven) electroretinogram responses until 29 dpf [47], and so these sampling times were also selected to represent stages in the functional maturation of rods. The *xops:eGFP* transgenic line is known to show rod-specific transgene expression throughout the zebrafish lifespan [26], and so was considered appropriate as source material for these analyses.

**Fig. 4:**
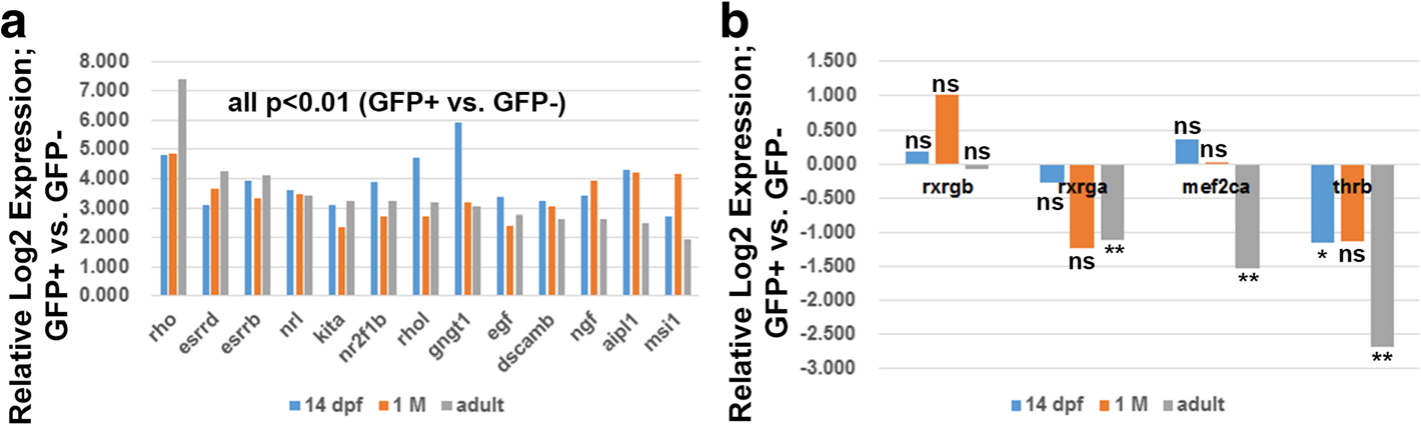
A. qPCR of selected transcripts enriched (**a**), or present (and not differentially expressed) or present (and depleted) in GFP+ (rod photoreceptor) cells (**b**), in larval retinas (14 dpf), juvenile retinas (1 M), and adult retinas. **, p < 0.01; *, p < 0.05; ns, not significantly differentially expressed, for GFP+ vs. GFP- (three biological replicates)

In general, the relative expression levels of selected rod-enriched transcripts within the GFP+ vs. GFP- cell populations were remarkably stable from larval through adult stages (Fig. 4a). However, *rho* transcripts appeared more highly enriched in rods of adult zebrafish than in rods of larval or juvenile zebrafish, suggesting that rods of adults may accumulate transcript at higher levels than rods of younger fish. *Rhol* (rhodopsin-like) transcripts showed the opposite trend (Fig. 4a), consistent with the recent findings of Morrow et al. [48], who detected limited expression of *rhol* in adult retina. Other transcripts more highly enriched in the rods of younger fish were *gngt1*, *ngf*, and *aipl1*. Transcripts present, or present but significantly depleted in adult rods, were less consistent over the lifespan; notable were *rxrga* and *mef2ca*, which were not differentially expressed in GFP+ vs. GFP- cells of larvae or juveniles, but were significantly downregulated (depleted) in rods of older zebrafish (Fig. 4b). Rod-specific functions for the encoded nuclear hormone receptor may be distinctive for rods of younger vs. older zebrafish.

**Fig. 5:**
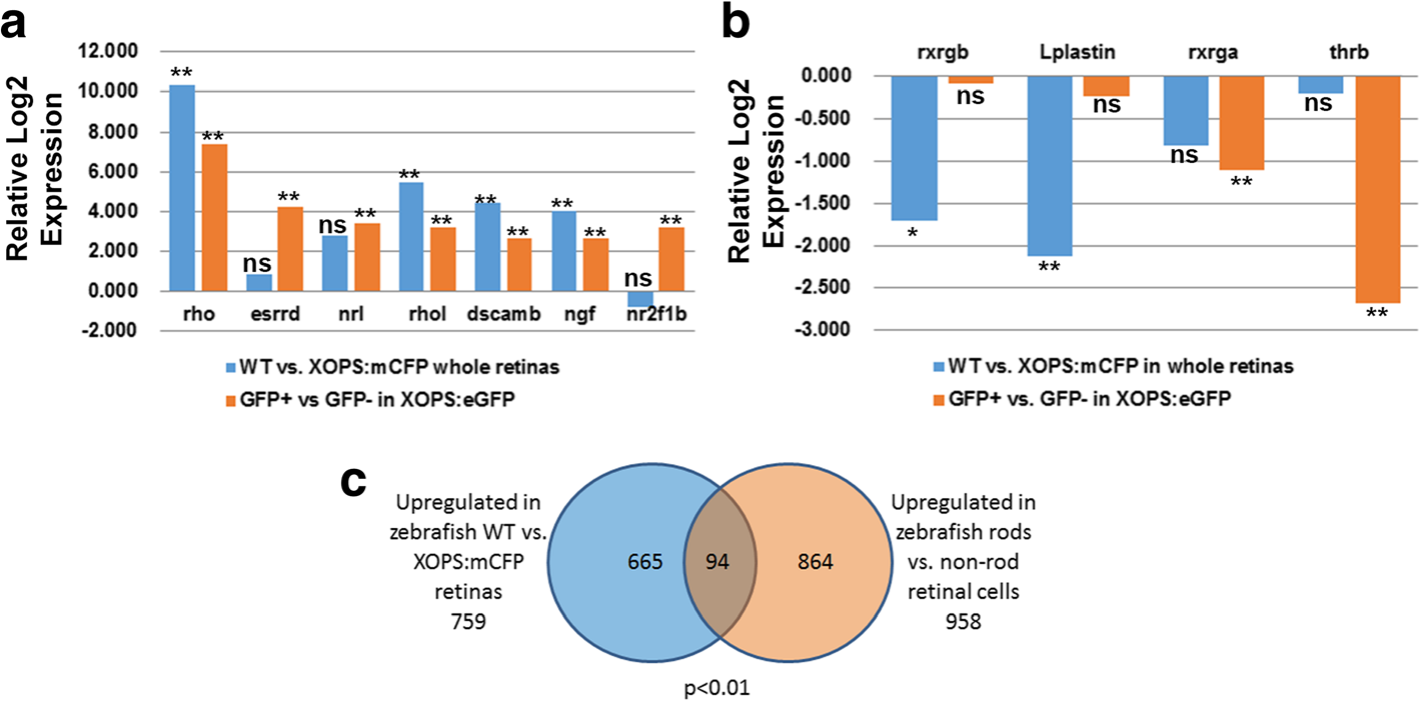
qPCR of selected transcripts enriched (**a**), or present (and not differentially expressed) or present (and depleted) in GFP+ (rod photoreceptor) cells (**b**), and relative expression in WT vs. *xops:mCFP* retinas. **, p < 0.01; *, p < 0.05; ns, not significantly differentially expressed for GFP+ vs. GFP- cells, or for WT vs. *xops:mCFP* retinas (three biological replicates). **c** Numbers of unique transcripts upregulated in GFP+ vs. GFP- retinal cells (present study), those upregulated in WT vs. *xops:mCFP* retinas [37], at p < 0.01, and those shared by both sets

We next measured expression (by qPCR) of selected transcripts in whole retinas obtained from WT zebrafish and from *xops:mCFP* zebrafish, which show a chronic rod degeneration that stimulates proliferation of a rod precursor population [37]. We anticipated that transcripts identified in the present study as rod-enriched, would be upregulated in WT retinas (containing mature rods) vs. *xops:mCFP* retinas (not containing mature rods). This was true for *rho*, *rhol*, *dscamb*, and *ngf*, but not true for *esrrd*, *nrl*, and *nr2f1b*, which were not differentially expressed in WT vs. *xops:mCFP* retinas (Fig. 5a). It is possible that the latter genes may have other retinal functions in the response to the chronic loss of rods. We also tested two transcripts present (but not enriched) in rods, *rxrgb* and *Lplastin*, and these were both significantly differentially downregulated in WT vs. *xops:mCFP* retinas (Fig. 5b), again suggestive of roles in response to chronic loss of rods. Two transcripts depleted in rods, and known to be expressed in cones in zebrafish or other model organisms, *rxrga* and *thrb* [45, 49–51], were not differentially expressed in WT vs. *xops:mCFP* retinas, consistent with their likely predominant localization to cones, which are unaffected in the *xops:mCFP* zebrafish [9]. Differentially expressed genes in WT vs. *xops:mCFP* retinas have previously been identified using microarray (GEO Acc # GSE22221) [37], allowing a deeper comparison of the two datasets. Using a cutoff of *p* < 0.01 for both datasets returned 94 shared entries (Fig. 5c; Additional file 1: Table S1), including known photoreceptor genes *aanat1*, *pde6a*, *rom1a*, and *rom1b*. We believe the number shared by the datasets is limited to 94 transcripts is in part due to the incomplete representation of zebrafish transcripts on the Agilent chip used for the microarray study.

**Fig. 6:**
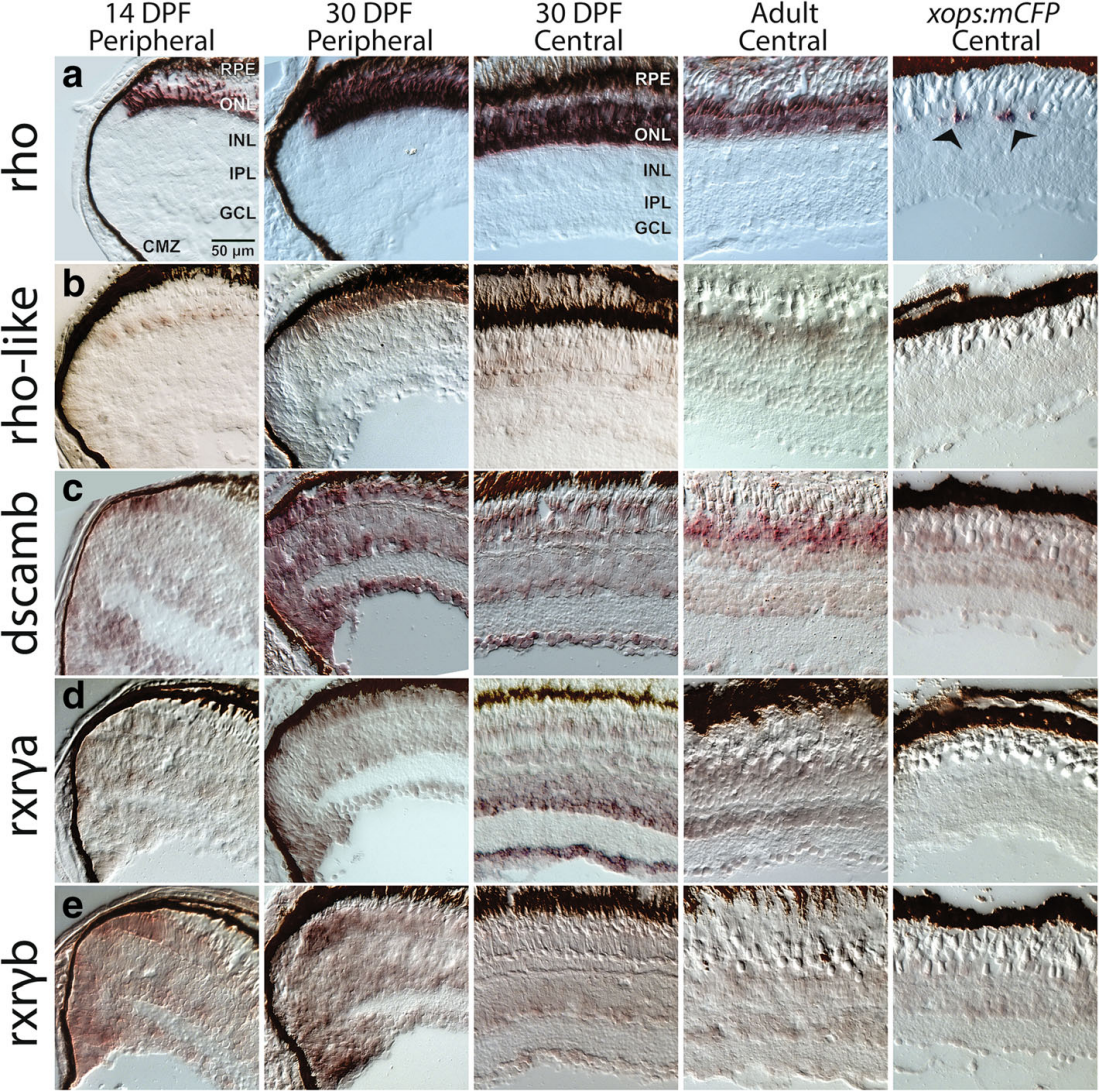
In situ hybridization for transcripts enriched (*rho, rhol, dscamb*) or present (*rxrga, rxrgb*) in rods of adult zebrafish, using tissues sampled from larvae (14 days post-fertilization; 14 DPF), juveniles (30 DPF), and adult WT fish, and from *xops:mCFP* transgenics, which show a chronic rod degeneration [9]. **a** Expression patterns for *rho*. Arrows in last panel show degenerating rods in *xops:mCFP* retina. **b** Expression patterns for *rhol*. **c** Expression patterns for *dscamb*. **d** Expression patterns for *rxrga*. **e** Expression patterns for *rxrgb*. DPF, days post-fertilization; CMZ, ciliary marginal zone; RPE, retinal pigmented epithelium; ONL, outer nuclear layer (photoreceptor layer); INL, inner nuclear layer; IPL, inner plexiform layer; GCL, ganglion cell layer. Scale bar (applies to all) = 50 μm

Selected transcripts were further examined by in situ hybridization to visualize spatial expression patterns. For these studies we selected *rhol* and *dscamb* as rod-enriched transcripts, and *rxrga* and *rxrgb* as transcripts present in rods (Fig. 6). The expression pattern of *rho* is shown for reference as an example of a rod-exclusive hybridization pattern (Fig. 6a). *Rhol* (*rh1–2*) was previously detected as a second *rho* gene in zebrafish [52] and other teleost fish [48], with demonstrated phototransduction functions and expression in the photoreceptor layer [48]. In the present study we have confirmed that *rhol* is expressed (and enriched) in rods, based upon RNA-seq and qPCR of purified rods (Fig. 6b). In tissues sampled at 14 dpf, *rhol* was expressed in the photoreceptor layer, in a subset of cells matching the distribution of rods, but restricted primarily to the peripheral retina. This predominantly peripheral pattern was evident in retinas sampled at 1 month, and in adult retina, where only very weak expression was detected in central retina (Fig. 6b). In retinas of the *xops:mCFP* line that displays rod degeneration, *rhol* was not detected by in situ hybridization, even though some developing/dying *rho*-expressing cells are present (Fig. 6b). In rods that express *rhol*, the timing of expression may be delayed as compared with *rho* (*rho* is first expressed embryonically, while *rhol* is first expressed in larvae [52]), and rods of *xops:mCFP* zebrafish may simply not survive long enough to express *rhol*.

*Dscamb* is one of two zebrafish orthologues of mammalian *Dscam*. Mammalian Dscam encodes a homophilic cell adhesion molecule with numerous roles in retinal cell patterning and refinement of circuitry [53, 54], but is not expressed in mouse rods [55] (although *dscamlike1* is expressed in mouse rods; [56]. In larval and juvenile zebrafish retinas, *dscamb* was expressed in some cells of the outer nuclear layer (photoreceptor layer; ONL), in a pattern consistent with identities of rods and possibly a subset of cones, and was also seen in the inner nuclear layer (INL) and ganglion cell layer (GCL) in a pattern suggestive of amacrine cells (Fig. 6c). Expression of *dscamb* in adult zebrafish retinas showed similar patterns (Fig. 6c). The *xops:mCFP* retinas showed apparently greatly reduced expression in the photoreceptor layer, consistent with *dscamb* localization to rods (Fig. 6c).

In larval and juvenile zebrafish, *rxrga* and *rxrgb* were both diffusely expressed in all retinal cellular layers, and more strongly localized to the far peripheral photoreceptor layer and the stem/progenitor cell-containing CMZ (Fig. 6d, e). The former pattern suggests transient higher expression in newly-generated photoreceptors, consistent with our previous report of expression of transient expression of *rxrga* in photoreceptors of zebrafish embryos [57]. Juvenile and adult zebrafish WT retinas, and those of *xops:mCFP* fish, both showed a diffuse pattern throughout all cellular layers, although the juvenile samples showed more pronounced expression of *rxrga* within the GCL and inner INL (Fig. 6d, e). These findings are consistent with the lack of significant enrichment of these transcripts in rods of adult zebrafish as detected by RNA-seq and qPCR (Fig. 3).

### Rod photoreceptor transcripts in regenerated retina

**Fig. 7:**
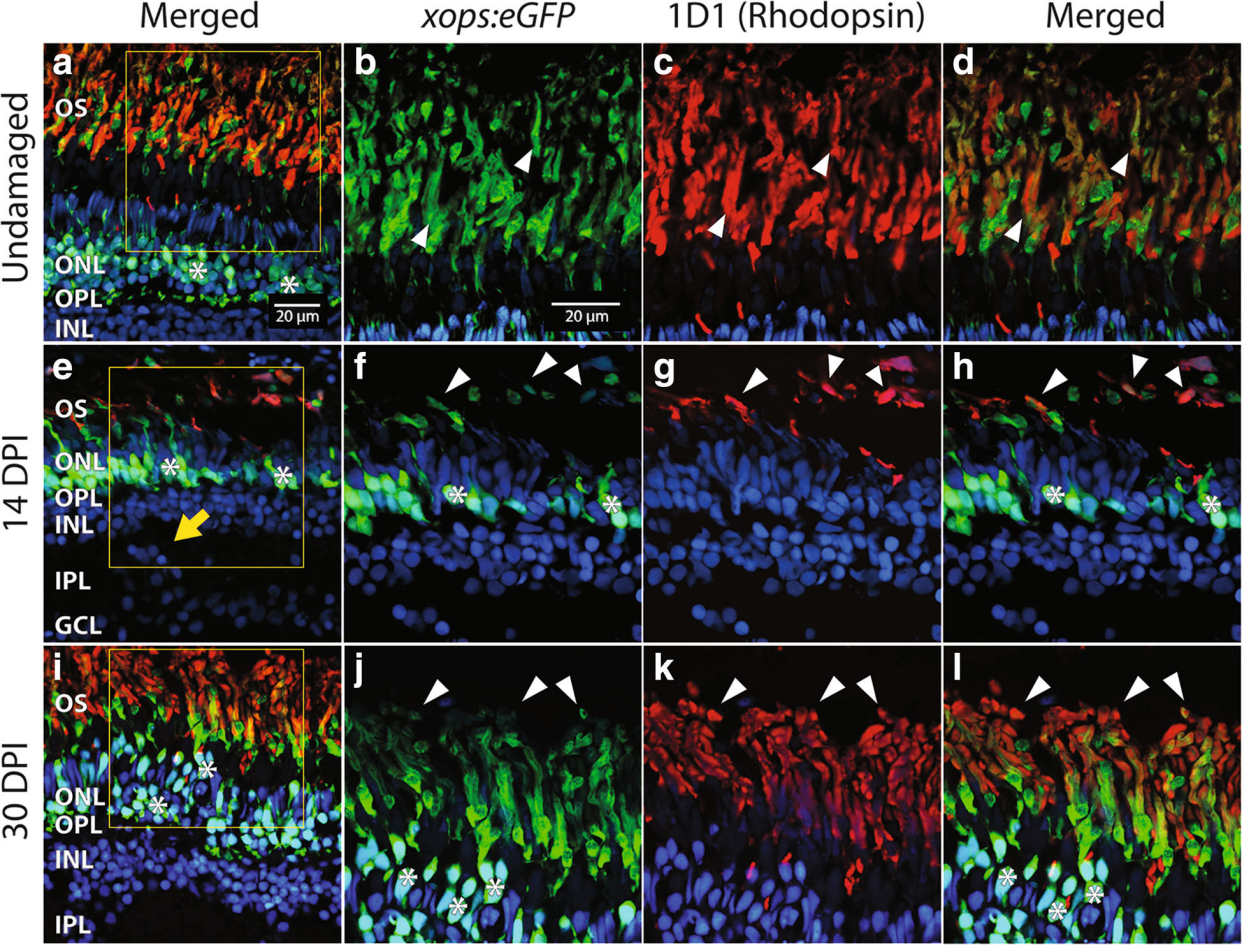
Regenerated rods in *xops:eGFP* transgenic zebrafish. **a** Undamaged retina, showing GFP+ (green); rod cell bodies in the outer nuclear layer (ONL) and GFP+, rhodopsin (1D1, magenta) outer segments (OS). Section is counterstained with DAPI (nuclei; blue). Asterisks (*) highlight some of the GFP+, DAPI+ cell bodies in the outer nuclear layer (ONL). Boxed area is shown at higher magnification in (**b-d**). **b-d**. Higher magnification views of outer segments of GFP imaging channel only (**b**), rhodopsin only (**c**), and a merged image (**d**), to show colabeling of outer segments (OS; some are highlighted by arrowheads). **e-h** Regenerated retina processed 14 days after injury (14 dpi), low magnification view with GPF, rhodopsin, and DAPI (**e**), and higher magnification views of GFP (**f**), rhodopsin (**g**), and merged image (**h**), to show colabeling of outer segments in regenerated retina (some are highlighted by arrowheads). Arrow in **e** shows example of histological error, with a cluster of DAPI+ cell bodies misplaced in the inner plexiform layer (IPL). **i-l** Regenerated retina processed 30 days after injury (30 dpi), low magnification view with GPF, rhodopsin, and DAPI (**i**), and higher magnification views of GFP (**j**), rhodopsin (**k**), and merged image (**l**), to show colabeling of outer segments in regenerated retina (some are highlighted by arrowheads). Boxed area in (**i**) is shown at higher magnification in (**j-l**). In **b-d**, **f-h**, and **j-k**, note that GFP fluorescence can be brighter than the corresponding 1D1 fluorescence, or vice-versa, although all OS show colabeling. Scale bar in **a** (applies to **a, e, i**) = 20 μm. Scale bar in **b** (applies to **b-d, f-h, j-l**) = 20 μm. OPL, outer plexiform layer; INL, inner nuclear layer; GCL, ganglion cell layer

The zebrafish regenerates a functional retina following widespread damage due to intravitreal injection of the neurotoxin ouabain [11]. However, regenerated fish retinas display histological errors such as neuronal cell bodies present in plexiform layers [14, 15] (and see Fig. 7), and disruptions of two-dimensional patterning [58, 59]. Although microarray and other analyses have revealed transcriptional changes in whole retina in response to damage [60] and accompanying the proliferative response specifically of Müller glia [60–62], the molecular signatures of identified, regenerated retinal neurons have never been compared with those of native, undamaged retinal neurons. We sampled regenerated retinas at 14 dpi, a time when all retinal layers are known to be re-established, but with some histological errors and very thin plexiform layers [11]. Another set of regenerated retinas was sampled at 30 dpi, when plexiform layers have expanded, but histological errors remain. The fidelity of *xops:eGFP* reporter expression as rod-specific in regenerated retina has not, to our knowledge, been established. Therefore we processed retinal cryosections for visualization of the eGFP reporter together with staining by the 1D1 (anti-rhodopsin; [40]) monoclonal antibody, using confocal microscopy. GFP+ profiles were observed in the ONL of control retinas, and of 14 dpi and 30 dpi retinas, and were associated with DAPI+ nuclei (Fig. 7a–i, asterisks). This localization to the ONL is consistent with their identities as rods. Furthermore, 1D1+ outer segments in control and regenerated retina were co-labeled with GFP (Fig. 7b-d, f-h, j-l, arrowheads), suggesting that regenerated, GFP+ cells in the *xops:eGFP* zebrafish retina corresponded to regenerated rods.

**Fig. 8:**
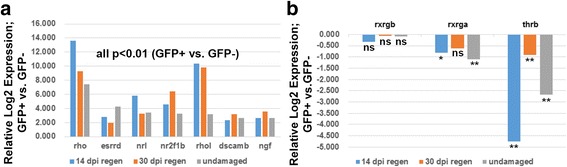
qPCR of selected transcripts enriched (**a**), or present (and not differentially expressed) or present (and depleted) in GFP+ (rod photoreceptor) cells (**b**), in regenerated retinas at 14 days post-injury (dpi), 30 dpi, and in undamaged retinas. **, p < 0.01; *, p < 0.05; ns, not significantly differentially expressed, for GFP+ vs. GFP- (three biological replicates)

Regenerated *xops:eGFP* retinas were dissociated, FACS-sorted, and subjected to qPCR of selected transcripts. Transcripts that were detected as significantly rod-enriched in undamaged retinas were also significantly rod-enriched in regenerated retinas at 14 and 30 dpi (Fig. 8a), providing an initial indication that regenerated rods are similar at the transcript level as the undamaged rods. Both *rho* and *rhol* were more highly enriched in the regenerating rods, and *nrl* was more highly enriched at 14 dpi (Fig. 8a). *Rxrgb* was detected, but not differentially expressed, in all samples, while *rxrga* was significantly depleted in rods of undamaged retina and at 14 dpi, but not 30 dpi (Fig. 8b). *Thrb*, important for determination of red-sensitive cones [51] was detected, but highly significantly depleted in all samples (Fig. 8b). Together these findings suggest that the rod transcriptome in regenerated retina possibly carries a molecular signature similar to that of undamaged rods.

## Discussion

We report for the first time, transcripts enriched, present, and depleted in rod photoreceptors of the adult zebrafish retina, now available as a resource for other investigators with interests in rod health, structure, function, and neurogenesis. The dataset was validated by qPCR of 45 transcripts, and many transcripts present in rods were not previously recognized as rod-enriched. Analysis of FACS-sorted fluorescent rods from transgenic zebrafish appears to be an excellent approach for expanding our knowledge of rod biology, and in the future may be applied to other photoreceptor subpopulations [63], since there are numerous transgenic tools available that selectively fluorescently label specific cone subtypes [64, 65].

The rod transcriptome appears to be remarkably stable over the zebrafish lifespan, at least for the rod-enriched transcripts studied in this manner, and at the sampling times used. The rod population of adult zebrafish, which includes the rods generated larvally – these rods are nearly as old as the zebrafish themselves – as well as the many rods that accumulated through adulthood, likely carries a molecular signature similar to that of the newly-generated rods of larval retina. The potential exceptions are the transcripts encoding the visual pigment proteins themselves, *rho* and *rhol*. *Rho* is more abundant in the rods from older fish, where the rod population includes many older rods. However, *rhol* is more abundant in those of younger fish. Interestingly, the peak spectral sensitivity of *rhol* is shifted 5 nm shorter than that of *rho* [48], although it is not known whether this difference is meaningful in the visual environment of zebrafish when rods are utilized. It is possible that the higher levels of *rhol* in rods of younger zebrafish are important for a visually-mediated behavior such as a prey capture strategy, that is different in larval/juvenile vs. adult zebrafish [66]. Extending the unbiased RNA-seq approach for the study of rod transcripts over the zebrafish lifespan may reveal other functional changes.

We used two approaches to evaluate rod transcripts in situations where the zebrafish retina responds to rod damage. In the first approach, we analyzed selected rod-enriched, and rod-depleted genes in WT retinas vs. those with chronic loss and attempted replacement of rods (*xops:mCFP*) [9]. Some of these rod-enriched transcripts were upregulated in WT retinas vs. *xops:mCFP*, consistent with the lack of mature rods in the *xops:mCFP* retinas. However, some were not, pointing to alternative roles for these transcripts in some aspect of the response to chronic damage, for example in the environment of high levels of cell death, or in upregulation of rod precursor proliferation. The second approach was to analyze selected rod-enriched, and rod-depleted genes following widespread retinal damage and a regeneration period. We found that transcripts enriched in undamaged, native rods, also were enriched in regenerated rods. Again the extension of the unbiased RNA-seq approach is likely to be even more illuminating, but this initial result suggests that regenerated rods do not differ in expression of a set of selected transcripts in comparison with undamaged, native rods. This finding implies that regenerated retinal neurons may not carry with them alternative molecular signatures, and likely recover their distinctive functions [67].

## Conclusions

We report the generation and validation of an RNA-seq dataset describing the rod transcriptome of the zebrafish. This transcriptome appears stable across the zebrafish lifespan, and similar in regenerated rods as compared with undamaged rods. Future applications of this study include comparative photoreceptor transcriptomics (rods vs. each cone subtype), and comparative analysis with transcriptome information available from other model organisms including mouse [68], as well as from stem cell-derived human retinal organoids [69]. Such studies have potential to reveal further distinctions of cones vs. rods, and distinctions among vertebrates that may resolve questions of vertebrate photoreceptor evolution [25, 70–72].

## Additional file

Additional file 1: Supplemental **Figure S1.** A. Multidimensional scaling (MDS) plot to visualize the level of similarity among the eight samples (four GFP+ and four GFP-) analyzed by RNA-seq. B. Average log counts per million (CPM) as a function of biological coefficient of variation (BCV), indicating a trend in dispersion associated with expression. C. Smear plot highlighting (red) differentially expressed transcripts at FDR <0.05. **Table S1.** Transcripts detected as significantly upregulated (*p* <0.01) in GFP+ vs. GFP- retinal cells of *xops:eGFP* zebrafish (“rod enriched”), and also detected as significantly upregulated (*p* <0.01) in WT vs. *xops:mCFP* whole retinas. (DOCX 262 kb)

## Abbreviations

CGZ: Circumferential germinal zone
CMZ: Ciliary marginal zone
DPF: Days post-fertilization
DPI: Days post-injury
FACS: Fluorescence-Activated Cell Sorting
GCL: Ganglion cell layer
GO: Gene ontology
INL: Inner nuclear layer
ONL: Outer nuclear layer
WT: Wild-type
