# Supplementary material for: Transcripts within rod photoreceptors of the Zebrafish retina
Source: BMC Genomics. 2018 Feb 8;19:127. doi: 10.1186/s12864-018-4499-y (PMC5806438; doi:10.1186/s12864-018-4499-y)
Supplement: Supplementary file 1 — Supplemental Figure S1. A. Multidimensional scaling (MDS) plot to visualize the level of similarity among the eight samples (four GFP+ and four GFP-) analyzed by RNA-seq. B. Average log counts per million (CPM) as a function of biological coefficient of variation (BCV), indicating a trend in dispersion associated with expression. C. Smear plot highlighting (red) differentially expressed transcripts at FDR <0.05. Table S1. Transcripts detected as significantly upregulated (p <0.01) in GFP+ vs. GFP- retinal cells of xops:eGFP zebrafish (“rod enriched”), and also detected as significantly upregulated (p <0.01) in WT vs. xops:mCFP whole retinas. (DOCX 262 kb) [file 12864_2018_4499_MOESM1_ESM.docx]

**Additional Files for**

**
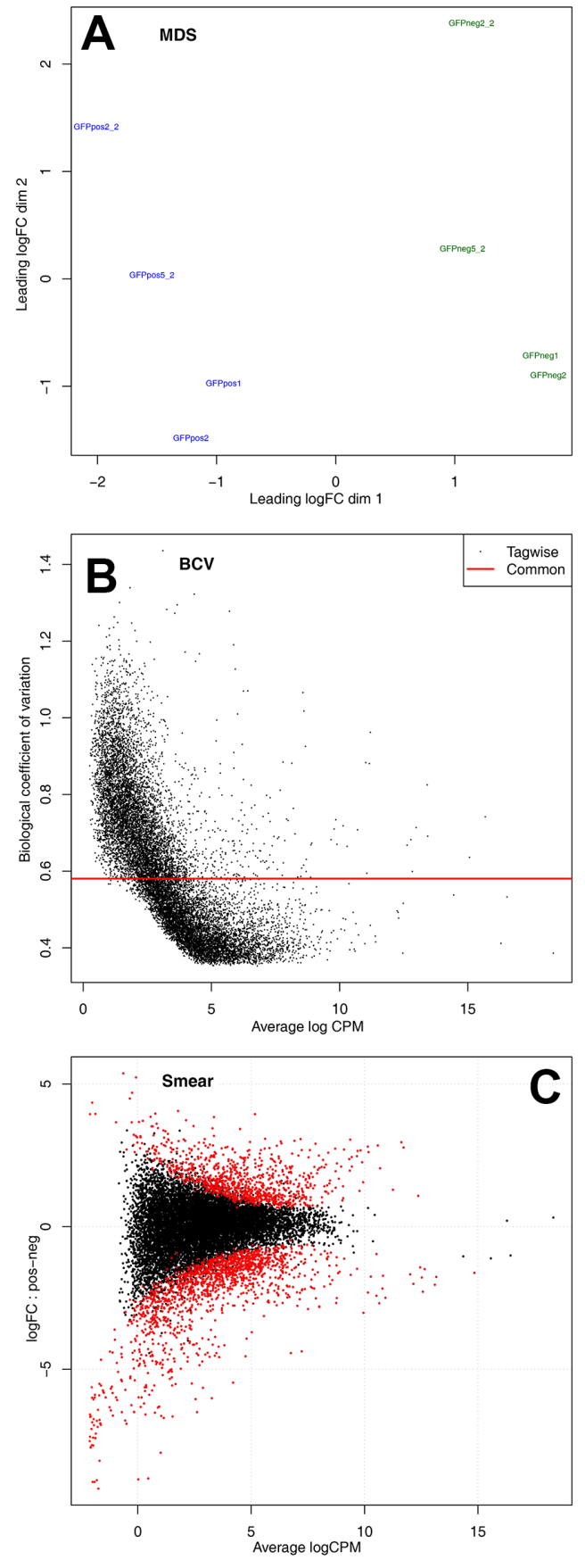
“Transcripts within Rod Photoreceptors of the Zebrafish Retina” by Sun et al.**

**Supplemental Figure S1.** A. Multidimensional scaling (MDS) plot to visualize the level of similarity among the eight samples (four GFP+ and four GFP-) analyzed by RNA-seq. B.

Average log counts per million (CPM) as a function of biological coefficient of variation (BCV), indicating a trend in dispersion associated with expression. C. Smear plot highlighting (red) differentially expressed transcripts at FDR <0.05.

**Supplemental Table S1.** Transcripts detected as significantly upregulated (p<0.01) in GFP+ vs. GFP- retinal cells of *xops:eGFP* zebrafish (“rod enriched”), and also detected as significantly upregulated (p<0.01) in WT vs. *xops:mCFP* whole retinas.

| **Name** | **Description** |
| --- | --- |
| [aanat1](http://www.ensembl.org/danio_rerio/Gene/Summary?db=core;g=ENSDARG00000036567) | arylalkylamine N-acetyltransferase 1 [Source:ZFIN;Acc:ZDB-GENE-040329-1] |
| [agpat3](http://www.ensembl.org/danio_rerio/Gene/Summary?db=core;g=ENSDARG00000036549) | 1-acylglycerol-3-phosphate O-acyltransferase 3 [Source:ZFIN;Acc:ZDB-GENE-040426-2765] |
| [arl13b](http://www.ensembl.org/danio_rerio/Gene/Summary?db=core;g=ENSDARG00000012763) | ADP-ribosylation factor-like 13b [Source:ZFIN;Acc:ZDB-GENE-021217-3] |
| [arl3l2](http://www.ensembl.org/danio_rerio/Gene/Summary?db=core;g=ENSDARG00000015404) | ADP-ribosylation factor-like 3, like 2 [Source:ZFIN;Acc:ZDB-GENE-040426-1678] |
| [atf4a](http://www.ensembl.org/danio_rerio/Gene/Summary?db=core;g=ENSDARG00000039515) | activating transcription factor 4a [Source:ZFIN;Acc:ZDB-GENE-040426-2340] |
| [atp6v0a1a](http://www.ensembl.org/danio_rerio/Gene/Summary?db=core;g=ENSDARG00000020847) | ATPase, H+ transporting, lysosomal V0 subunit a1a [Source:ZFIN;Acc:ZDB-GENE-030131-3027] |
| [bzw2](http://www.ensembl.org/danio_rerio/Gene/Summary?db=core;g=ENSDARG00000035918) | basic leucine zipper and W2 domains 2 [Source:ZFIN;Acc:ZDB-GENE-040426-746] |
| [c2h1orf27](http://www.ensembl.org/danio_rerio/Gene/Summary?db=core;g=ENSDARG00000042671) | c2h1orf27 homolog (H. sapiens) [Source:ZFIN;Acc:ZDB-GENE-080502-1] |
| [casz1](http://www.ensembl.org/danio_rerio/Gene/Summary?db=core;g=ENSDARG00000037030) | castor zinc finger 1 [Source:ZFIN;Acc:ZDB-GENE-060130-108] |
| [ccdc157](http://www.ensembl.org/danio_rerio/Gene/Summary?db=core;g=ENSDARG00000056279) | coiled-coil domain containing 157 [Source:ZFIN;Acc:ZDB-GENE-030616-155] |
| [cdc34a](http://www.ensembl.org/danio_rerio/Gene/Summary?db=core;g=ENSDARG00000069708) | cell division cycle 34 homolog (S. cerevisiae) a [Source:ZFIN;Acc:ZDB-GENE-040426-713] |
| [chchd2](http://www.ensembl.org/danio_rerio/Gene/Summary?db=core;g=ENSDARG00000059304) | coiled-coil-helix-coiled-coil-helix domain containing 2 [Source:ZFIN;Acc:ZDB-GENE-040426-1737] |
| [csnk1da](http://www.ensembl.org/danio_rerio/Gene/Summary?db=core;g=ENSDARG00000008370) | casein kinase 1, delta a [Source:ZFIN;Acc:ZDB-GENE-030131-825] |
| [cspp1b](http://www.ensembl.org/danio_rerio/Gene/Summary?db=core;g=ENSDARG00000091628) | centrosome and spindle pole associated protein 1b [Source:ZFIN;Acc:ZDB-GENE-070912-619] |
| [dnajc5ga](http://www.ensembl.org/danio_rerio/Gene/Summary?db=core;g=ENSDARG00000041896) | DnaJ (Hsp40) homolog, subfamily C, member 5 gamma a [Source:ZFIN;Acc:ZDB-GENE-030131-1583] |
| [dolpp1](http://www.ensembl.org/danio_rerio/Gene/Summary?db=core;g=ENSDARG00000036915) | dolichyldiphosphatase 1 [Source:ZFIN;Acc:ZDB-GENE-041024-11] |
| [efr3a](http://www.ensembl.org/danio_rerio/Gene/Summary?db=core;g=ENSDARG00000005163) | EFR3 homolog A (S. cerevisiae) [Source:ZFIN;Acc:ZDB-GENE-040426-1681] |
| [egf](http://www.ensembl.org/danio_rerio/Gene/Summary?db=core;g=ENSDARG00000052739) | epidermal growth factor [Source:ZFIN;Acc:ZDB-GENE-070922-1] |
| [elovl4b](http://www.ensembl.org/danio_rerio/Gene/Summary?db=core;g=ENSDARG00000027495) | ELOVL fatty acid elongase 4b [Source:ZFIN;Acc:ZDB-GENE-030131-7672] |
| [eno1b](http://www.ensembl.org/danio_rerio/Gene/Summary?db=core;g=ENSDARG00000013750) | enolase 1b, (alpha) [Source:ZFIN;Acc:ZDB-GENE-040426-1651] |
| [epb41l5](http://www.ensembl.org/danio_rerio/Gene/Summary?db=core;g=ENSDARG00000032324) | erythrocyte membrane protein band 4.1 like 5 [Source:ZFIN;Acc:ZDB-GENE-030616-450] |
| [faimb](http://www.ensembl.org/danio_rerio/Gene/Summary?db=core;g=ENSDARG00000020814) | Fas apoptotic inhibitory molecule b [Source:ZFIN;Acc:ZDB-GENE-040718-91] |
| [glcea](http://www.ensembl.org/danio_rerio/Gene/Summary?db=core;g=ENSDARG00000005095) | glucuronic acid epimerase a [Source:ZFIN;Acc:ZDB-GENE-040630-9] |
| [gngt1](http://www.ensembl.org/danio_rerio/Gene/Summary?db=core;g=ENSDARG00000035798) | guanine nucleotide binding protein (G protein), gamma transducing activity polypeptide 1 [Source:ZFIN;Acc:ZDB-GENE-030131-7596] |
| [gpsm2](http://www.ensembl.org/danio_rerio/Gene/Summary?db=core;g=ENSDARG00000017311) | G-protein signaling modulator 2 [Source:ZFIN;Acc:ZDB-GENE-040426-1169] |
| [guca1a](http://www.ensembl.org/danio_rerio/Gene/Summary?db=core;g=ENSDARG00000010454) | guanylate cyclase activator 1A [Source:ZFIN;Acc:ZDB-GENE-011128-5] |
| [hacd2](http://www.ensembl.org/danio_rerio/Gene/Summary?db=core;g=ENSDARG00000014806) | 3-hydroxyacyl-CoA dehydratase 2 [Source:ZFIN;Acc:ZDB-GENE-030131-6053] |
| [hrsp12](http://www.ensembl.org/danio_rerio/Gene/Summary?db=core;g=ENSDARG00000035882) | heat-responsive protein 12 [Source:ZFIN;Acc:ZDB-GENE-040718-315] |
| [htt](http://www.ensembl.org/danio_rerio/Gene/Summary?db=core;g=ENSDARG00000052866) | huntingtin [Source:ZFIN;Acc:ZDB-GENE-990415-131] |
| [icmt](http://www.ensembl.org/danio_rerio/Gene/Summary?db=core;g=ENSDARG00000020241) | isoprenylcysteine carboxyl methyltransferase [Source:ZFIN;Acc:ZDB-GENE-050417-90] |
| [id2b](http://www.ensembl.org/danio_rerio/Gene/Summary?db=core;g=ENSDARG00000029544) | inhibitor of DNA binding 2, dominant negative helix-loop-helix protein, b [Source:ZFIN;Acc:ZDB-GENE-030131-15] |
| [kita](http://www.ensembl.org/danio_rerio/Gene/Summary?db=core;g=ENSDARG00000043317) | v-kit Hardy-Zuckerman 4 feline sarcoma viral oncogene homolog a [Source:ZFIN;Acc:ZDB-GENE-980526-464] |
| [kri1](http://www.ensembl.org/danio_rerio/Gene/Summary?db=core;g=ENSDARG00000075380) | KRI1 homolog [Source:ZFIN;Acc:ZDB-GENE-040915-3] |
| [ldb3a](http://www.ensembl.org/danio_rerio/Gene/Summary?db=core;g=ENSDARG00000056322) | LIM domain binding 3a [Source:ZFIN;Acc:ZDB-GENE-040121-6] |
| [lingo1b](http://www.ensembl.org/danio_rerio/Gene/Summary?db=core;g=ENSDARG00000035899) | leucine rich repeat and Ig domain containing 1b [Source:ZFIN;Acc:ZDB-GENE-040912-136] |
| [lrrc57](http://www.ensembl.org/danio_rerio/Gene/Summary?db=core;g=ENSDARG00000043938) | leucine rich repeat containing 57 [Source:ZFIN;Acc:ZDB-GENE-040718-372] |
| [lypla2](http://www.ensembl.org/danio_rerio/Gene/Summary?db=core;g=ENSDARG00000053656) | lysophospholipase II [Source:ZFIN;Acc:ZDB-GENE-040426-1715] |
| [mak](http://www.ensembl.org/danio_rerio/Gene/Summary?db=core;g=ENSDARG00000059287) | male germ cell-associated kinase [Source:ZFIN;Acc:ZDB-GENE-030131-7279] |
| [mao](http://www.ensembl.org/danio_rerio/Gene/Summary?db=core;g=ENSDARG00000023712) | monoamine oxidase [Source:ZFIN;Acc:ZDB-GENE-040329-3] |
| [map3k14a](http://www.ensembl.org/danio_rerio/Gene/Summary?db=core;g=ENSDARG00000074060) | mitogen-activated protein kinase kinase kinase 14a [Source:ZFIN;Acc:ZDB-GENE-120215-80] |
| [mbip](http://www.ensembl.org/danio_rerio/Gene/Summary?db=core;g=ENSDARG00000019364) | MAP3K12 binding inhibitory protein 1 [Source:ZFIN;Acc:ZDB-GENE-060810-43] |
| [MCUR1](http://www.ensembl.org/danio_rerio/Gene/Summary?db=core;g=ENSDARG00000016964) | mitochondrial calcium uniporter regulator 1 [Source:HGNC Symbol;Acc:HGNC:21097] |
| [meis1b](http://www.ensembl.org/danio_rerio/Gene/Summary?db=core;g=ENSDARG00000012078) | Meis homeobox 1 b [Source:ZFIN;Acc:ZDB-GENE-020122-1] |
| [mest](http://www.ensembl.org/danio_rerio/Gene/Summary?db=core;g=ENSDARG00000032319) | mesoderm specific transcript [Source:ZFIN;Acc:ZDB-GENE-991111-5] |
| [mknk2a](http://www.ensembl.org/danio_rerio/Gene/Summary?db=core;g=ENSDARG00000011373) | MAP kinase interacting serine/threonine kinase 2a [Source:ZFIN;Acc:ZDB-GENE-030131-6099] |
| [msi1](http://www.ensembl.org/danio_rerio/Gene/Summary?db=core;g=ENSDARG00000010710) | musashi RNA-binding protein 1 [Source:ZFIN;Acc:ZDB-GENE-050320-86] |
| [nfs1](http://www.ensembl.org/danio_rerio/Gene/Summary?db=core;g=ENSDARG00000062237) | NFS1 cysteine desulfurase [Source:ZFIN;Acc:ZDB-GENE-060405-1] |
| [ngfb](http://www.ensembl.org/danio_rerio/Gene/Summary?db=core;g=ENSDARG00000014050) | nerve growth factor b (beta polypeptide) [Source:ZFIN;Acc:ZDB-GENE-000629-2] |
| [nr2f1b](http://www.ensembl.org/danio_rerio/Gene/Summary?db=core;g=ENSDARG00000017168) | nuclear receptor subfamily 2, group F, member 1b [Source:ZFIN;Acc:ZDB-GENE-040426-1438] |
| [nucb2b](http://www.ensembl.org/danio_rerio/Gene/Summary?db=core;g=ENSDARG00000036291) | nucleobindin 2b [Source:ZFIN;Acc:ZDB-GENE-030131-4775] |
| [ophn1](http://www.ensembl.org/danio_rerio/Gene/Summary?db=core;g=ENSDARG00000035420) | oligophrenin 1 [Source:ZFIN;Acc:ZDB-GENE-040718-464] |
| [oxr1b](http://www.ensembl.org/danio_rerio/Gene/Summary?db=core;g=ENSDARG00000063310) | oxidation resistance 1b [Source:ZFIN;Acc:ZDB-GENE-030131-2438] |
| [pdca](http://www.ensembl.org/danio_rerio/Gene/Summary?db=core;g=ENSDARG00000011886) | phosducin a [Source:ZFIN;Acc:ZDB-GENE-031023-1] |
| [pde6a](http://www.ensembl.org/danio_rerio/Gene/Summary?db=core;g=ENSDARG00000000380) | phosphodiesterase 6A, cGMP-specific, rod, alpha [Source:ZFIN;Acc:ZDB-GENE-030616-42] |
| [PDE6H](http://www.ensembl.org/danio_rerio/Gene/Summary?db=core;g=ENSDARG00000056791) | zgc:112320 [Source:ZFIN;Acc:ZDB-GENE-050522-144] |
| [pelo](http://www.ensembl.org/danio_rerio/Gene/Summary?db=core;g=ENSDARG00000055477) | pelota homolog (Drosophila) [Source:ZFIN;Acc:ZDB-GENE-040426-1074] |
| [pex5](http://www.ensembl.org/danio_rerio/Gene/Summary?db=core;g=ENSDARG00000070654) | peroxisomal biogenesis factor 5 [Source:ZFIN;Acc:ZDB-GENE-040426-981] |
| [pisd](http://www.ensembl.org/danio_rerio/Gene/Summary?db=core;g=ENSDARG00000052462) | phosphatidylserine decarboxylase [Source:ZFIN;Acc:ZDB-GENE-061215-46] |
| [plcd1b](http://www.ensembl.org/danio_rerio/Gene/Summary?db=core;g=ENSDARG00000034080) | phospholipase C, delta 1b [Source:ZFIN;Acc:ZDB-GENE-030131-9435] |
| [ppdpfa](http://www.ensembl.org/danio_rerio/Gene/Summary?db=core;g=ENSDARG00000007682) | pancreatic progenitor cell differentiation and proliferation factor a [Source:ZFIN;Acc:ZDB-GENE-030219-204] |
| [ppp1cab](http://www.ensembl.org/danio_rerio/Gene/Summary?db=core;g=ENSDARG00000071566) | protein phosphatase 1, catalytic subunit, alpha isozyme b [Source:ZFIN;Acc:ZDB-GENE-030131-5512] |
| [ppp2r5d](http://www.ensembl.org/danio_rerio/Gene/Summary?db=core;g=ENSDARG00000014428) | protein phosphatase 2, regulatory subunit B', delta [Source:ZFIN;Acc:ZDB-GENE-040426-2568] |
| [prom1b](http://www.ensembl.org/danio_rerio/Gene/Summary?db=core;g=ENSDARG00000034007) | prominin 1 b [Source:ZFIN;Acc:ZDB-GENE-031003-1] |
| [prr12b](http://www.ensembl.org/danio_rerio/Gene/Summary?db=core;g=ENSDARG00000075849) | proline rich 12b [Source:ZFIN;Acc:ZDB-GENE-130625-2] |
| [PTP4A3](http://www.ensembl.org/danio_rerio/Gene/Summary?db=core;g=ENSDARG00000054814) | protein tyrosine phosphatase type IVA, member 3 [Source:HGNC Symbol;Acc:HGNC:9636] |
| [ptpn11b](http://www.ensembl.org/danio_rerio/Gene/Summary?db=core;g=ENSDARG00000012340) | protein tyrosine phosphatase, non-receptor type 11, b [Source:ZFIN;Acc:ZDB-GENE-040426-1158] |
| [pygb](http://www.ensembl.org/danio_rerio/Gene/Summary?db=core;g=ENSDARG00000002021) | phosphorylase, glycogen; brain [Source:ZFIN;Acc:ZDB-GENE-040928-2] |
| [rcbtb1](http://www.ensembl.org/danio_rerio/Gene/Summary?db=core;g=ENSDARG00000036645) | regulator of chromosome condensation (RCC1) and BTB (POZ) domain containing protein 1 [Source:ZFIN;Acc:ZDB-GENE-030131-7951] |
| [rcl1](http://www.ensembl.org/danio_rerio/Gene/Summary?db=core;g=ENSDARG00000038312) | RNA terminal phosphate cyclase-like 1 [Source:ZFIN;Acc:ZDB-GENE-040930-11] |
| [rgs9b](http://www.ensembl.org/danio_rerio/Gene/Summary?db=core;g=ENSDARG00000045156) | regulator of G-protein signaling 9b [Source:ZFIN;Acc:ZDB-GENE-040426-1708] |
| [rom1a](http://www.ensembl.org/danio_rerio/Gene/Summary?db=core;g=ENSDARG00000019752) | retinal outer segment membrane protein 1a [Source:ZFIN;Acc:ZDB-GENE-040426-1765] |
| [rom1b](http://www.ensembl.org/danio_rerio/Gene/Summary?db=core;g=ENSDARG00000026926) | retinal outer segment membrane protein 1b [Source:ZFIN;Acc:ZDB-GENE-040426-1073] |
| [sebox](http://www.ensembl.org/danio_rerio/Gene/Summary?db=core;g=ENSDARG00000042526) | SEBOX homeobox [Source:ZFIN;Acc:ZDB-GENE-021206-4] |
| [sf3b2](http://www.ensembl.org/danio_rerio/Gene/Summary?db=core;g=ENSDARG00000018049) | splicing factor 3b, subunit 2 [Source:ZFIN;Acc:ZDB-GENE-070928-1] |
| [sh3glb2b](http://www.ensembl.org/danio_rerio/Gene/Summary?db=core;g=ENSDARG00000035470) | SH3-domain GRB2-like endophilin B2b [Source:ZFIN;Acc:ZDB-GENE-040426-833] |
| [si:ch211-195b15.7](http://www.ensembl.org/danio_rerio/Gene/Summary?db=core;g=ENSDARG00000017579) | si:ch211-195b15.7 [Source:ZFIN;Acc:ZDB-GENE-131121-183] |
| [si:dkey-45l12.1](http://www.ensembl.org/danio_rerio/Gene/Summary?db=core;g=ENSDARG00000097472) | si:dkey-45l12.1 [Source:ZFIN;Acc:ZDB-GENE-131121-316] |
| [si:dkey-7n6.2](http://www.ensembl.org/danio_rerio/Gene/Summary?db=core;g=ENSDARG00000094602) | si:dkey-7n6.2 [Source:ZFIN;Acc:ZDB-GENE-091204-262] |
| [sirt2](http://www.ensembl.org/danio_rerio/Gene/Summary?db=core;g=ENSDARG00000011488) | sirtuin 2 (silent mating type information regulation 2, homolog) 2 (S. cerevisiae) [Source:ZFIN;Acc:ZDB-GENE-030131-1028] |
| [slc16a6b](http://www.ensembl.org/danio_rerio/Gene/Summary?db=core;g=ENSDARG00000060246) | solute carrier family 16, member 6b [Source:ZFIN;Acc:ZDB-GENE-110208-3] |
| [smad3b](http://www.ensembl.org/danio_rerio/Gene/Summary?db=core;g=ENSDARG00000010207) | SMAD family member 3b [Source:ZFIN;Acc:ZDB-GENE-030128-4] |
| [spata6l](http://www.ensembl.org/danio_rerio/Gene/Summary?db=core;g=ENSDARG00000004874) | spermatogenesis associated 6-like [Source:ZFIN;Acc:ZDB-GENE-040426-1369] |
| [TDRD7 (1 of many)](http://www.ensembl.org/danio_rerio/Gene/Summary?db=core;g=ENSDARG00000077523) | tudor domain containing 7 [Source:HGNC Symbol;Acc:HGNC:30831] |
| [tegt](http://www.ensembl.org/danio_rerio/Gene/Summary?db=core;g=ENSDARG00000077934) | testis enhanced gene transcript (BAX inhibitor 1) [Source:ZFIN;Acc:ZDB-GENE-030826-10] |
| [tmem218](http://www.ensembl.org/danio_rerio/Gene/Summary?db=core;g=ENSDARG00000027129) | transmembrane protein 218 [Source:ZFIN;Acc:ZDB-GENE-040426-1232] |
| [trim71](http://www.ensembl.org/danio_rerio/Gene/Summary?db=core;g=ENSDARG00000075593) | tripartite motif containing 71, E3 ubiquitin protein ligase [Source:ZFIN;Acc:ZDB-GENE-040128-1] |
| [tsc1a](http://www.ensembl.org/danio_rerio/Gene/Summary?db=core;g=ENSDARG00000026048) | tuberous sclerosis 1a [Source:ZFIN;Acc:ZDB-GENE-030131-9111] |
| [uap1](http://www.ensembl.org/danio_rerio/Gene/Summary?db=core;g=ENSDARG00000052170) | UDP-N-acetylglucosamine pyrophosphorylase 1 [Source:ZFIN;Acc:ZDB-GENE-030131-1233] |
| [unc119.2](http://www.ensembl.org/danio_rerio/Gene/Summary?db=core;g=ENSDARG00000004459) | unc-119 lipid binding chaperone B homolog 2 [Source:ZFIN;Acc:ZDB-GENE-030131-7635] |
| [vps36](http://www.ensembl.org/danio_rerio/Gene/Summary?db=core;g=ENSDARG00000057021) | vacuolar protein sorting 36 homolog (S. cerevisiae) [Source:ZFIN;Acc:ZDB-GENE-030131-7753] |
| [zdhhc16b](http://www.ensembl.org/danio_rerio/Gene/Summary?db=core;g=ENSDARG00000015989) | zinc finger, DHHC-type containing 16b [Source:ZFIN;Acc:ZDB-GENE-040426-1301] |
| [zgc:103625](http://www.ensembl.org/danio_rerio/Gene/Summary?db=core;g=ENSDARG00000038770) | zgc:103625 [Source:ZFIN;Acc:ZDB-GENE-040912-116] |
| [zgc:56231](http://www.ensembl.org/danio_rerio/Gene/Summary?db=core;g=ENSDARG00000010332) | zgc:56231 [Source:ZFIN;Acc:ZDB-GENE-040426-1914] |
| [zgc:77752](http://www.ensembl.org/danio_rerio/Gene/Summary?db=core;g=ENSDARG00000042387) | zgc:77752 [Source:ZFIN;Acc:ZDB-GENE-040426-1867] |
